# Identification of Potential Binders of *Mtb* Universal Stress Protein (Rv1636) Through an *in silico* Approach and Insights Into Compound Selection for Experimental Validation

**DOI:** 10.3389/fmolb.2021.599221

**Published:** 2021-05-03

**Authors:** Sohini Chakraborti, Moubani Chakraborty, Avipsa Bose, Narayanaswamy Srinivasan, Sandhya S. Visweswariah

**Affiliations:** ^1^Molecular Biophysics Unit, Indian Institute of Science, Bengaluru, India; ^2^Department of Molecular Reproduction, Development and Genetics, Indian Institute of Science, Bengaluru, India

**Keywords:** virtual screening, molecular docking, universal stress protein, Rv1636, anti-tubercular compounds, computational drug discovery, MM-GBSA, experimental insights

## Abstract

Millions of deaths caused by *Mycobacterium tuberculosis* (*Mtb*) are reported worldwide every year. Treatment of tuberculosis (TB) involves the use of multiple antibiotics over a prolonged period. However, the emergence of resistance leading to multidrug-resistant TB (MDR-TB) and extensively drug-resistant TB (XDR-TB) is the most challenging aspect of TB treatment. Therefore, there is a constant need to search for novel therapeutic strategies that could tackle the growing problem of drug resistance. One such strategy could be perturbing the functions of novel targets in *Mtb*, such as universal stress protein (USP, Rv1636), which binds to cAMP with a higher affinity than ATP. Orthologs of these proteins are conserved in all mycobacteria and act as “sink” for cAMP, facilitating the availability of this second messenger for signaling when required. Here, we have used the cAMP-bound crystal structure of USP from *Mycobacterium smegmatis*, a closely related homolog of *Mtb*, to conduct a structure-guided hunt for potential binders of Rv1636, primarily employing molecular docking approach. A library of 1.9 million compounds was subjected to virtual screening to obtain an initial set of ~2,000 hits. An integrative strategy that uses the available experimental data and consensus indications from other computational analyses has been employed to prioritize 22 potential binders of Rv1636 for experimental validations. Binding affinities of a few compounds among the 22 prioritized compounds were tested through microscale thermophoresis assays, and two compounds of natural origin showed promising binding affinities with Rv1636. We believe that this study provides an important initial guidance to medicinal chemists and biochemists to synthesize and test an enriched set of compounds that have the potential to inhibit *Mtb* USP (Rv1636), thereby aiding the development of novel antitubercular lead candidates.

## Introduction

Tuberculosis (TB), a contagious and airborne disease caused by the pathogen *Mycobacterium tuberculosis (Mtb)*, was one of the top 10 causes of deaths worldwide in the year 2019 as per World Health Organization (WHO) global TB report 2020 (WHO, 2020). It is also a major cause of deaths in HIV patients and deaths due to antimicrobial resistance. The WHO has identified a gap of over USD 1.2 billion per year for TB research in its global TB report 2019 (WHO, 2019). The economic distress due to the ongoing coronavirus disease 2019 (COVID-19) pandemic is further threatening to stall or reverse the progress that has been achieved (WHO, 2020). Therefore, the reduction in TB disease burden calls for the scientific community's attention to contribute toward finding rational solutions for improving the current scenario. While isoniazid, rifampicin, pyrazinamide, and ethambutol are effective against drug-susceptible TB (DST-TB), multidrug resistant-TB (MDR-TB) infections do not respond to at least isoniazid and rifampicin. Extensively drug-resistant TB (XDR-TB) is a more serious problem that is resistant not only to the two key first-line drugs (isoniazid and rifampicin) but also to fluoroquinolones and second-line aminoglycosides leaving only limited options of treatment with reserved third-line drugs that possess higher toxicities. Totally drug-resistant TB (TDR-TB) correspond to the most severe forms of the infection, where all the first and second line of drugs fail to produce any response (Bahuguna and Rawat, 2020). As of August 2020, there were 22 drugs in different stages of clinical trials, including 13 new compounds: BTZ-043, delpazolid, GSK-3036656, macozinone, OPC-167832, Q203, SQ109, SPR720, sutezolid, TBAJ-876, TBA-7371, TBI-166, and TBI-223. Six approved antimicrobial drugs, namely, clofazimine, levofloxacin, linezolid, moxifloxacin, rifampicin (high dose), and rifapentine, are also undergoing trials for repurposing against TB. Host-directed therapies such as auronofin, CC-11050 (AMG 634), and everolimus are also being evaluated (WHO, 2020). Understanding the mechanism of action (MOA) of these drugs is important to formulate novel drug regimens well-tolerated by patients with comorbidities, improve cost effectiveness, and reduce therapy time. The MOA of some of the anti-TB drugs currently in the clinical pipeline has been reviewed elsewhere (Shetye et al., 2020). The introduction of promising novel anti-TB drugs like bedaquiline and delamanid in the last decade has brought new rays of hope (Koul et al., 2007; Lakshmanan and Xavier, 2013; Xavier and Lakshmanan, 2014). Unfortunately, the emergence of resistance to these drugs has also been reported (Bloemberg et al., 2015; Ghodousi et al., 2019; Nieto Ramirez et al., 2020). This calls for a constant effort to devise strategies for combating the emerging global problem of drug resistance.

An effective way to tackle drug resistance can be by targeting novel proteins that are involved in critical biological pathways in the organism and have not been targeted in the past, such as the cAMP signaling pathways. The presence of cAMP in both slow- and fast-growing mycobacteria was first noticed in the 1970s (Lowrie et al., 1975, 1979; Padh and Venkitasubramanian, 1976). These studies also showed that a large portion of cAMP (~80% for *Mycobacterium microti*) was secreted in the culture medium (Lowrie et al., 1975). Lowrie et al. first showed the involvement of this molecule in the pathogenicity of mycobacteria. They observed a correlation between the increase in cAMP levels and the absence of phagolysosomal fusion within macrophages. This increase in cAMP was not seen upon infection with latex beads or heat-killed mycobacteria (Lowrie et al., 1975, 1979). The genome sequences of mycobacteria further endorse the importance of cAMP in their survival and virulence. Compared to other bacteria, these organisms encode a wide array of adenylyl cyclases: 16 in *M. tuberculosis* and 31 in *M. marinum—*in stark contrast to the one adenylyl cyclase of *Escherichia coli* (Cole et al., 1998; Stinear et al., 2008). *M. tuberculosis* also encodes 11 cAMP binding proteins, further emphasizing the significance of the second messenger in the organism (Shenoy and Visweswariah, 2006). Studies with *Mycobacterium smegmatis* showed that synthesis of cAMP was not an exclusive characteristic of slow-growing, pathogenic mycobacteria. cAMP levels in *M. smegmatis* were found to be highest during its exponential phase along with a considerable amount of secretion (Dass et al., 2008). During the infection of host alveolar macrophages, cAMP levels inside host cells increase by several folds, possibly due to secretion of cAMP as a “toxin” by the bacteria. The different fates of pathogenic vs. non-pathogenic mycobacteria within host macrophages can also be explained by changes in host's cAMP levels—non-pathogenic *M. smegmatis* causing a sustained elevation of cAMP, whereas pathogenic *M. avium* causing a transient elevation (Yadav et al., 2004). Singh *et al*. also observed a similar “cAMP burst” in macrophages when infected with pathogenic *M. tuberculosis* H37Rv, in contrast to a constantly elevated cAMP level when infected with non-pathogenic *M. tuberculosis* H37Ra (Singh et al., 2012). Kalamidas et al. showed that cAMP interrupts phagosomal actin assembly and, thus, prevents fusion of lysosome with phagosome and its acidification (Kalamidas et al., 2006).

Previously, we reported that a significant fraction of intracellular cAMP is bound to a mycobacterial universal stress protein (USP), Rv1636, that is abundantly expressed in both slow-growing as well as fast-growing mycobacteria (Banerjee et al., 2015). Rv1636 could possibly act as a “sink” for cAMP and release these second messengers on demand to facilitate signaling processes when required. Thorough biochemical and thermodynamic characterization of Rv1636 was subsequently performed, and the crystal structure of *M. smegmatis* USP (MSMEG_3811, a close homolog of *Mtb* USP, Rv1636) bound to cAMP was determined (Banerjee et al., 2015). Presuming that cAMP is extremely crucial for the survival and virulence of *Mtb*, targeting Rv1636 with an inhibitor could perturb overall cAMP signaling in the pathogen leading to reduced virulence.

The available chemical space to search for a potential compound that might bind to a target of interest is huge and requires high throughout compound screenings. Computational screening pipelines serve as useful tools to rationally narrow down the chemical search space in a comparatively shorter time. Furthermore, careful design of virtual chemical libraries prior to screening also generally reduces the risk of failures of drug discovery programs triggered due to toxicity (Walters et al., 1998; Mohs and Greig, 2017). In the current study, we have used the crystal structure of cAMP-bound MSMEG_3811 (PDB code: 5AHW) (Banerjee et al., 2015) as a guide to derive important knowledge about critical protein–ligand interactions. A workflow primarily driven by *in silico* approach integrated with available experimental data helped us prioritize 22 compounds that have the potential to bind to *Mtb* USP (Rv1636). These compounds were identified by computationally screening large libraries of chemical compounds (~1.9 million), including synthetic and natural compounds. Two natural compounds identified from the virtual screening have shown promising results in *in vitro* experiments. Additionally, a library of approved drugs was screened virtually to identify potential drugs that can be repurposed against Rv1636. Therefore, this study provides many potential starting points for design, synthesis, and testing of a new class of antitubercular compounds that might bind Rv1636.

## Materials and Methods

**Figure 1:**
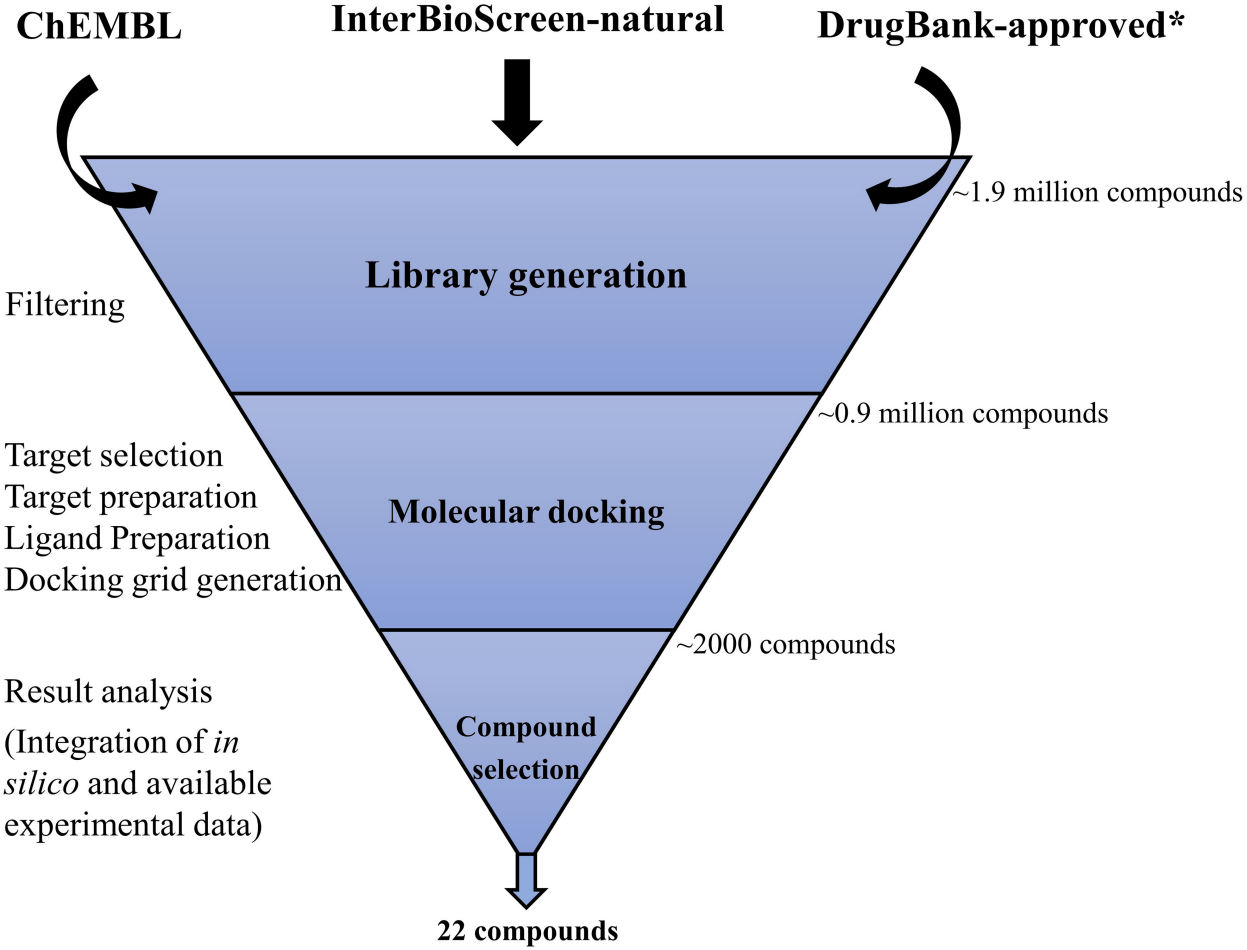
Virtual screening workflow adopted in this study. Approximately 1.9 million compounds were collated from three different libraries (ChEMBL, InterBioScreen-natural, and DrugBank-approved). ChEMBl and InterBioScreen-natural compound libraries were subjected to filtering to discard molecules that are less likely to be good drug candidates. A clean library of ~0.9 million compounds that includes the DrugBank-approved library was then subjected to hierarchical modes of docking simulations (HTVS, SP, XP). Nearly 2,000 hits obtained from this screening step were subjected to analyses to prioritize 22 promising compounds for further investigation as potential *Mtb* USP (Rv1636) binders. *The DrugBank-approved drugs library was not subjected to this filtering step as discussed in the text.

Our search for potential binders of *Mtb* USP involved a rigorous virtual screening workflow (Figure 1) comprising of four major steps, which are elaborated below.

### *In silico* Analyses

#### Target Structure Selection

It is known that protein binding site residues can show structural deviations in their ligand-bound state (holo) compared to the ligand-free (apo) state. Such structural deviations can alter the binding site's shape and volume, modulating the protein–ligand recognition pattern (Fradera et al., 2002; Cozzini et al., 2008; Clark et al., 2019). Earlier studies have shown that preformed protein binding sites in holo conformation are more likely to best distinguish between binders and non-binders in virtual screenings implemented through molecular docking protocols (McGovern and Shoichet, 2003; Rueda et al., 2010).

**Figure 2:**
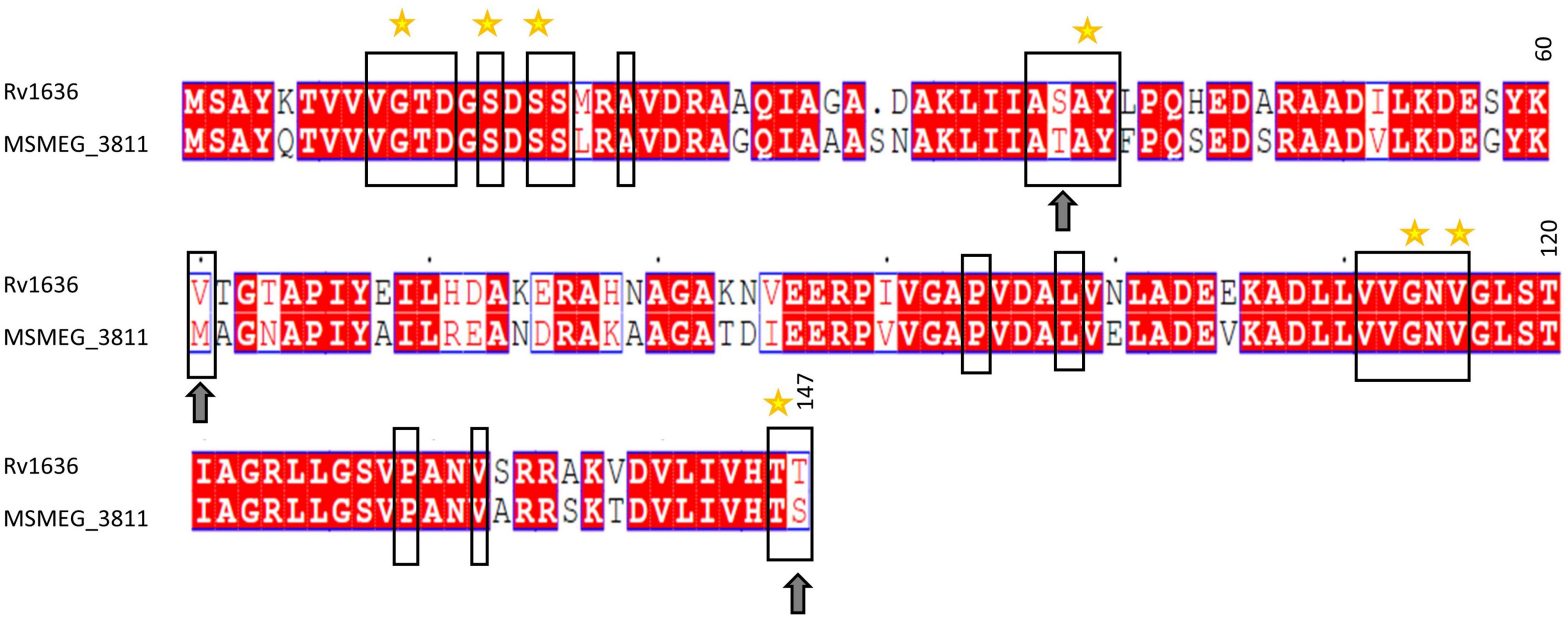
Full-length sequence alignment of Rv1636 (*Mtb* USP) and MSMEG_3811 (*M. smegmatis* USP). The sequences have been aligned using the EMBL-EBI online tool, EMBOSS (Madeira et al., 2019), and the alignment has been viewed using ESPript 3.0 (Robert and Gouet, 2014). The columns with identical residues are highlighted in red box. Similar residues are indicated in red font. The black rectangles enclose the binding site residues. The yellow stars are placed above the residues that are shown to be hydrogen bonded with cAMP in the crystal structure of PDB code: 5AHW. The arrows indicate non-identical but similar residues in the binding site. The numbers above the residues at the end of each block indicate the particular residue position in the sequence of *Mtb* USP (Rv1636).

The structure of any inhibitor/native ligand (cAMP)-bound *Mtb* USP (Rv1636; holo conformation) is currently not available in the Protein Databank (PDB) (Berman et al., 2000). The only experimentally determined structure of Rv1636 in the PDB is an apo crystal structure (PDB code: 1TQ8) (Rajashankar et al., 2004). However, a crystal structure of *M. smegmatis* USP (MSMEG_3811; PDB code: 5AHW, 2.15Å), which is a close homolog of Rv1636 (sequence identity: 70%; Figure 2) is available. The crystal structure of MSMEG_3811 is bound to the native ligand, cAMP. Comparative analysis of the cAMP binding site residues reveal that the binding sites of MSMEG_3811 and Rv1636 are highly conserved (Figure 2). Interestingly, overlay of the cAMP-bound MSMEG_3811 structure on to the apo Rv1636 structure revealed that a few binding sites residues show considerable backbone and side-chain deviations due to a shift of a stretch of residues (residues 117–146 in 5AHW) toward the cAMP binding pocket in the holo conformation as compared to the apo state (Supplementary Figure 1). Furthermore, when compared to the crystal structure 5AHW, residues equivalent to positions 44–64 are missing in the electron density map of all the chains of the crystal structure 1TQ8. One of these residues (Met61 in 5AHW, which is equivalent to Val60 in 1TQ8) lines the cAMP binding site and can thus influence the interaction profile of docked ligands. The electron density map of one of the chains (chain C) of 5AHW has no missing residues. Therefore, the crystal structure of holo MSMEG_3811 with a preformed pocket that hosts cAMP (with no missing residues in the binding site of chain C) is more suited than the apo Rv1636 structure to screen for potential binders that can target the cAMP-binding pocket of *Mtb* USP. Therefore, here, we have used chain C of 5AHW for the docking study. Importantly, our earlier studies indicated that cAMP exhibits comparable binding affinities with Rv1636 and MSMEG_3811 (Banerjee et al., 2015). Thus, a compound that binds to the cAMP binding site of MSMEG_3811 is likely to bind to Rv1636.

#### Target Structure Preparation

The reliability of the predictions from docking studies is largely dependent on the accuracy of the atomic coordinates of the input structures. For a structure determined by X-ray crystallography, a good fit of the atomic model to observed electron density ensures the reliability of the position of the atoms. Earlier studies revealed instances of overenthusiastic interpretation of ligand density (Deller and Rupp, 2015). Therefore, the quality of the ligand and binding site residues of the input structure (PDB code: 5AHW) was inspected using the EDIA (electron density score for individual atoms) tool (Meyder et al., 2017). The structure was also visually inspected against its electron density map. Supplementary Table 1 shows that the quality of the binding site residues and bound cAMP in the chain C of 5AHW is satisfactory.

The binding site of cAMP in MSMEG_3811 is away from the interface of the protomers. Thus, only one chain of the homo-multimeric protein was chosen for docking experiments (Supplementary Figure 2). In the chain C of 5AHW, Val113, a residue lining the cAMP binding pocket has been modeled with dual conformations; each conformer has an occupancy of 0.5, indicating that both these conformers have equal influences on modulating ligand interactions and, thus, can differentially influence the outcomes of virtual screening (Supplementary Figure 2). Hence, during target preparation, both the conformers of Val113 were considered by fixing the coordinates of each conformer of the residue one at a time in two separate protein models, hereafter referred to as conformer I and conformer II. To minimize the chances of missing potential hits favored by only one of the two conformers, we docked the ligand library against both the available conformers (I and II). Ligands that fit well to the binding sites of both the conformers could also be identified and prioritized for testing, as these ligands are likely to have higher chances of binding to the protein at the specified site.

Protein preparation wizard available in the Schrödinger software package was used for the target preparation (Sastry et al., 2013). Hydrogen atoms were added to the structure. Water molecules and other unwanted crystallization aids were deleted from the binding site. The protonation states of the bound ligand, cAMP, were generated using Epik (Shelley et al., 2007; Greenwood et al., 2010) at pH 7.0 ± 0.5, and the protein was prepared at pH 7.4. Hydrogen bonding network in the structure was subjected to optimization followed by a restrained minimization so that heavy atoms converge to RMSD 0.3 Å, and the hydrogen atoms were fully optimized. This was done to ensure that strains in the structure are relieved alongside full relaxation of the hydrogen bonding network.

#### Ligand Library Generation and Preparation

A library of 1.9 million compounds was generated by collating compounds from three databases: ChEMBL, version 24.1 (Mendez et al., 2018); InterBioScreen-natural compounds (downloaded in September 2018; https://www.ibscreen.com/natural-compounds); and DrugBank, version 5.1.1 (approved molecules) (Wishart et al., 2006). The compounds from the ChEMBL and InterBioScreen library were subjected to cleaning by using the (i) structural and (ii) molecular property filters offered by Canvas (Duan et al., 2010; Sastry et al., 2010). The structural filters aided in removing compounds following the Rapid Elimination of Swill (REOS) (Walters et al., 1998) and Pan-Assay Interference Compounds (PAINS) (Baell and Holloway, 2010) concepts to enrich the library with molecules that are less likely to be toxic and promiscuous. The molecular property filters eliminated compounds (with molecular weight >500 Da, hydrogen bond acceptor and donor count more than 10 and 5, respectively, and AlogP > 5) that are less likely to be successful oral drugs (Lipinski et al., 1997; Lipinski, 2000). From the DrugBank database, the subset of approved small-molecule drugs was included in our library. These molecules were not subjected to pre-filtering, as the known information on the safety and usages of these drugs could be exploited in prioritizing compounds for testing as discussed later. Finally, a clean library comprising of 0.9 million compounds was prepared by desalting and generating tautomers and stereoisomers at pH 7 ± 1 using the LigPrep module of Schrödinger package.

Additionally, we prepared a library of 14 compounds that demonstrated or were predicted to bind to Rv1636 through experimental or computational approaches, respectively. Two out of the 14 compounds include cAMP and ATP, where cAMP is known to bind to Rv1636 with a 10-fold higher affinity than ATP (Banerjee et al., 2015). cAMP and ATP served as the control compounds for our docking studies. The remaining 12 compounds include 10 polyphenolic compounds and 2 approved drugs (amikacin and kanamycin). We refer to the library of these 12 compounds as the secondary library. The 10 polyphenolic compounds were suggested to be potential binders of *Mtb* USP (Rv1636) by Aanandhi et al. (2014) based on their docking studies, where the compounds were docked at a site different from the cAMP binding site. We were interested in checking the possibility of binding of these compounds at the cAMP binding site. In a study by Sharma et al. (2016), it has been observed that Rv1636 is overexpressed in amikacin- and kanamycin-resistant *Mtb* isolates. They further performed docking studies to show that both the mentioned drugs have the potential to bind to the conserved USP domain of Rv1636. Docking of the control and secondary library of compounds was performed to ensure the validity of our protocol in reproducing the pose of the bound native ligand, cAMP, understand whether the results from our docking studies correlate with previously reported experimental binding affinities of cAMP and ATP toward MSMEG_3811, and compare the predicted binding affinities of the compounds in our primary library with those of the control and secondary library compounds.

#### Molecular Docking

Molecular docking of all the prepared chemical compounds was performed using Glide implemented in the virtual screening workflow (VSW) of Schrödinger software package (Friesner et al., 2004, 2006; Halgren et al., 2004). The grid box for docking the compounds was generated for both conformers I and II. Default settings in the Glide Receptor Grid Generation module were used for generating the two grid boxes enclosing the cAMP binding site in conformers I and II, which involve specifying the centroid of the bound cAMP as center of the grid box and choosing a box size that accommodates ligands similar to the size of the bound ligand. The van der Waals radii scaling factor for non-polar atoms of the protein was kept at 1.0 with a partial charge cut-off of 0.25. The percentage of output compounds from each stage of the hierarchical VSW protocol was specified in such a way so that not more than 1,000 top-scoring compounds were reported in the hit list after the final stage of screening the ChEMBL library. Similarly, the initial number of virtual hits obtained from the screening of the InterBioScreen-natural compound and DrugBank-approved drugs libraries were restricted to 50 and 20 top scoring compounds, respectively.

The hierarchical docking modes in VSW include the following stages: (i) high throughput virtual screening (HTVS), (ii) standard precision (SP), and (iii) extra precision (XP). The first stage performs HTVS, which is the fastest of the three stages and trades sampling exhaustiveness for higher speed. The ligands that are retained are passed on to the second stage, which performs SP docking. The Glide SP docking performs more exhaustive sampling than HTVS stage. Both HTVS and SP docking use the same scoring function (SP GlideScore) to rank order the ligand poses. This score is a “softer” function that aims to minimize false negatives during the virtual screening of a large database of compounds. The ligands that survive after the SP docking stage are then passed on to the third stage, XP docking, for a more rigorous sampling. The XP docking uses a “harder” scoring function that penalizes poses that violate expected physical chemistry principles, such as large desolvation of polar and charged groups. The third stage in the VSW reduces the false positives that SP docking lets through. Thus, the three stages of screening lead to rational funneling of a large library of compounds to a small set of candidate ligands ranked on their predicted ability to bind to the specified conformation of the protein of interest at a given site (Friesner et al., 2004, 2006; Halgren et al., 2004).

#### Compound Selection

From an initial hit list of ~2,000 compounds (~1,000 for each conformer), 22 compounds were selected for experimental testing (18 compounds from the ChEMBL library, 2 each from the InterBioScreen-natural and DrugBank-approved drug libraries). MMGBSA (implemented in Prime v3 of Schrödinger software package) calculations were performed on all the initial hits (~2,000 compounds; ~1,000 initial hits from each of the two conformers) to estimate the relative binding affinities of these ligands in the implicit solvent model against the respective conformer (I and II) of the protein. The VSGB solvent model was used, which employs the variable-dielectric generalized Born model, incorporating a residue-dependent effect, where the solvent is water. While the Glide dock scores are based on empirical scoring functions that distinguish binders from non-binders, Prime-MMGBSA is a physics-based method that computes relative binding free energies (dG_bind_) of bound and unbound molecules as per Equation 1, where E_complex_ is the minimized energy of the protein–ligand complex, and the E_ligand_ and E_receptor_ are the individual minimized energies in uncomplexed form. The absolute values calculated are not necessarily in agreement with experimental binding affinities. However, it has been shown earlier that ranking of ligands based on MMGBSA dG_bind_ scores agree reasonably with experimental binding energies and outperform empirical docking scores, especially in case of congeneric series of ligands (Lyne et al., 2006). These scores could serve as one of the guiding parameters for prioritizing analogous compounds while testing in an experimental setting. The docked poses of the ligands obtained from Glide-XP docking (final stage of VSW) served as the starting ligand structures for the Prime-MMGBSA calculations. The prepared protein structure for each of the two conformers used for docking the ligand library was taken as the input protein structure for the Prime-MMGBSA calculations. While the ligands' docked poses were subjected to relaxation, the protein atoms were kept rigid during the Prime-MMGBSA calculations.

(1) $$\begin{array}{l} dG_{bind}={\text{E}}_{\text{complex}}-\left({\text{E}}_{\text{ligand}}+{\text{E}}_{\text{receptor}}\right) \end{array}$$

From the initial hit list of ~2,000 compounds obtained from the ChEMBL library, 100 top-scoring compounds based on docking scores were prioritized for further analysis. The poses and interaction profiles of each compound were visually scrutinized to ensure that the docked compounds fit well into the desired binding pocket and most of the important binding site residues (such as Ala40, Gly10, Ser14, Ser16, Gly114, Val116, Thr146) are engaged in hydrogen-bond interactions with the docked compounds. The mentioned residues are hydrogen bonded with bound cAMP in the crystal structure (PDB code: 5AHW; Supplementary Figure 3). While the residues Ala40, Gly10, Gly114, and Val116 establish hydrogen bonds with cAMP through backbone carbonyl oxygen or amide nitrogen or both, the remaining residues are engaged in side-chain-mediated hydrogen bonding with cAMP. It has been shown earlier that mutation of Gly10 and Gly114 (which corresponds to Gly113 in Rv1636) to Thr and Ala, respectively, significantly compromise the binding of cAMP and ATP to MSMEG_3811 (Banerjee et al., 2015). Therefore, engagement of these residues in interactions with other compounds might inhibit cAMP binding to the protein.

In addition, available information on the bioactivity of the shortlisted compounds was fetched from ChEMBL and or PubChem (Kim et al., 2018). The compounds that are already known to be effective against tuberculosis infection were assigned a higher priority for testing and designated as “biased set” compounds. The remaining compounds (“Blind set”) were chosen based on chemical diversity (as indicated by “Tanimoto coefficient”) to ensure that representative compounds from each cluster of chemical compounds are tested in an experimental setup. The Canvas module available with Schrödinger software package was used to calculate 2D Tanimoto coefficient (Syuib et al., 2013) and subsequently for the chemical diversity analysis.

From the virtual screening of InterBioScreen-natural compound library, 50 top scoring hits were subjected to MMGBSA calculations, and interaction profile analysis was performed in a similar way as mentioned for the ChEMBL library. Two compounds were selected for testing from this library. Two out of the 20 approved drugs as obtained from screening the DrugBank library were selected based on the consensus docking results against conformers I and II followed by interaction profile analysis coupled with analysis of the data pertaining to known primary targets of the compounds as available in DrugBank. Besides, curcumin from the secondary library was selected for testing.

Since docking scores predicted approximate binding affinities between the protein and ligands and Prime-MMGBSA dG_bind_ scores are approximate relative binding energies between the bound and unbound state of the molecules, more negative scores indicate the possibility of stronger binding.

OPLS3e force field (Roos et al., 2019) was used throughout the entire computational study.

The non-covalent interactions between the protein and the docked compounds were visualized in Maestro GUI available with Schrödinger suite of programs. The geometric criteria used for the detection of these interactions are presented in Supplementary Table 2. The sketches of the chemical compounds provided in Table 1 are made using the 2D sketcher implemented in Maestro GUI (Schrödinger, LLC, New York). The figures of protein–ligand complexes were generated using Maestro GUI and PyMOL (Schrödinger, LLC).

**Table 1:** Results of molecular docking and Prime-MMGBSA calculation of 22 shortlisted candidates.

**Sl. No**.	**Compound**	**Docking score** **(kcal/mol)**	**Prime-MMGBSA dG_**bind**_** **score (kcal/mol)**	Interacting residues^*^
1	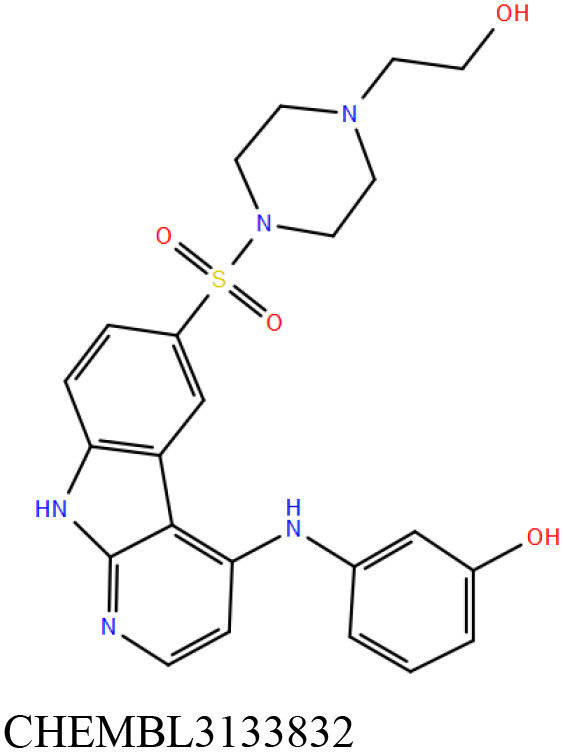	−11.8	−77.1	V9, **G10**, T11, D12, S17, A20, A38, T39, **A40**, Y41, F42, K60, M61, A62, P95, L99, V113, **G114**, N115, **V116**, G117, L118, G123, G127, S128, **V129**, P130, T146
2	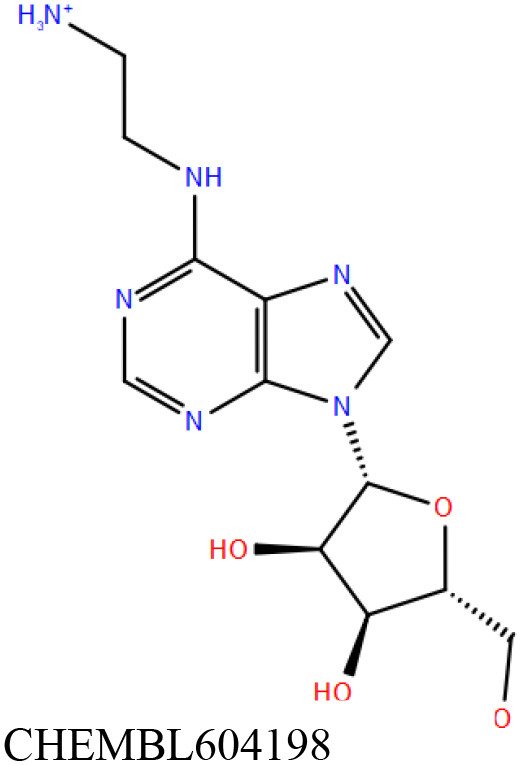	−11.8	−61.0	V9, **G10**, T11, D12, S14, S16, S17, A20, A38, T39, **A40**, Y41, F42, E57, M61, A62, A94, P95, L99, V112, V113, **G114**, N115, **V116**, G117, L118, S128, V129, P130, V133, T146
3	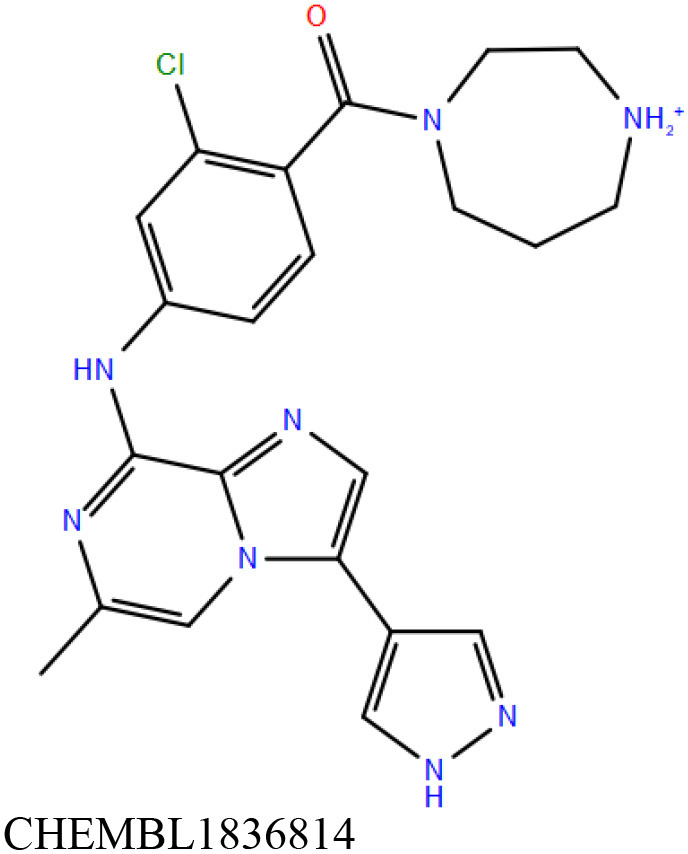	−11.6	−68.9	V9, G10, T11, D12, S17, A20, A38, T39, **A40**, Y41, F42, E57, K60, M61, A62, I67, V91, G93, A94, P95, A98, L99, V113, **G114**, N115, V116, V129, P130
4	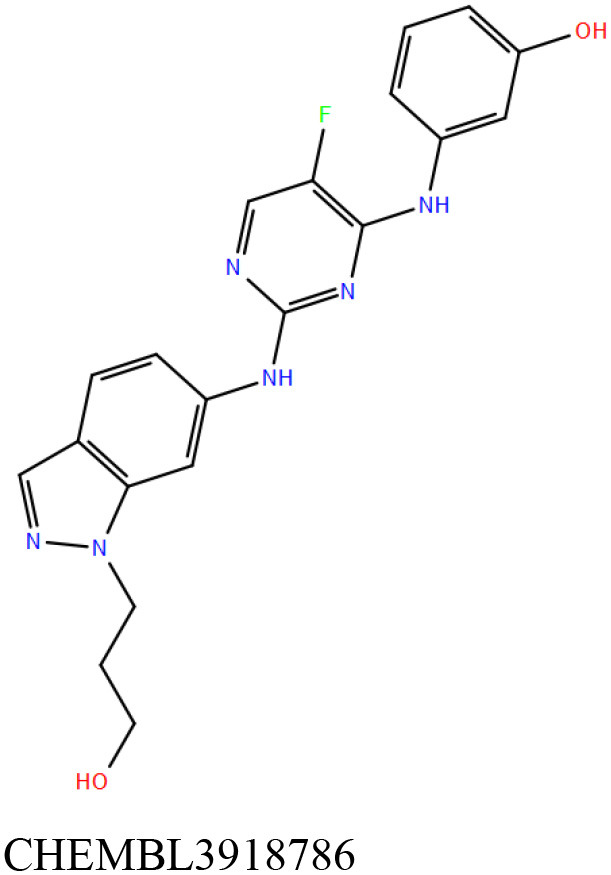	−11.1	−70.5	V9, **G10**, T11, **D12**, S14, S16, S17, A20, V21, A38, T39, **A40**, Y41, F42, E57, K60, M61, **A62**, G63, P95, L99, V113, G114, N115, V116, V129, P130, T146
5	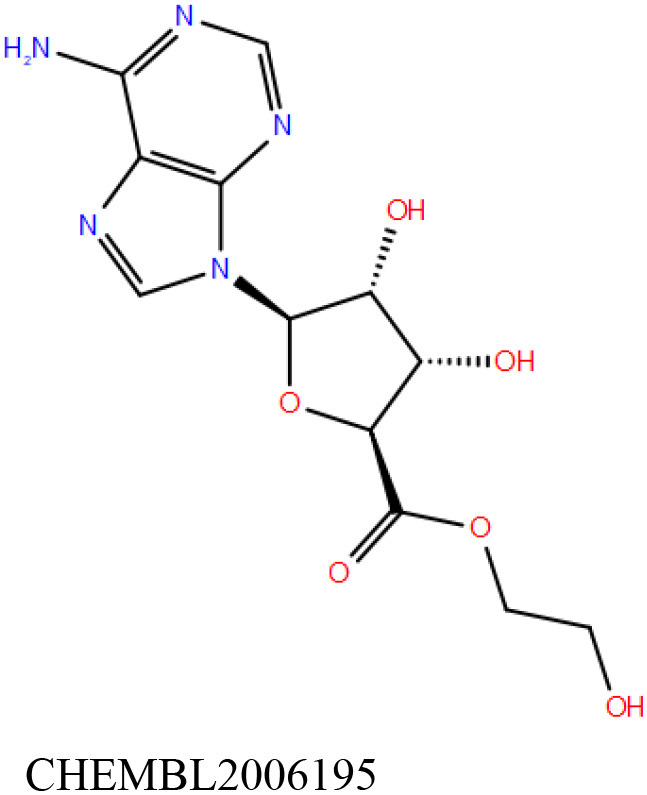	−11.1	−70.3	V9, **G10**, T11, D12, S14, S16, S17, A20, A38, T39, **A40**, Y41, M61, P95, L99, V112, V113, **G114**, N115, **V116**, G117, L118, S128, **V129**, P130, T146
6	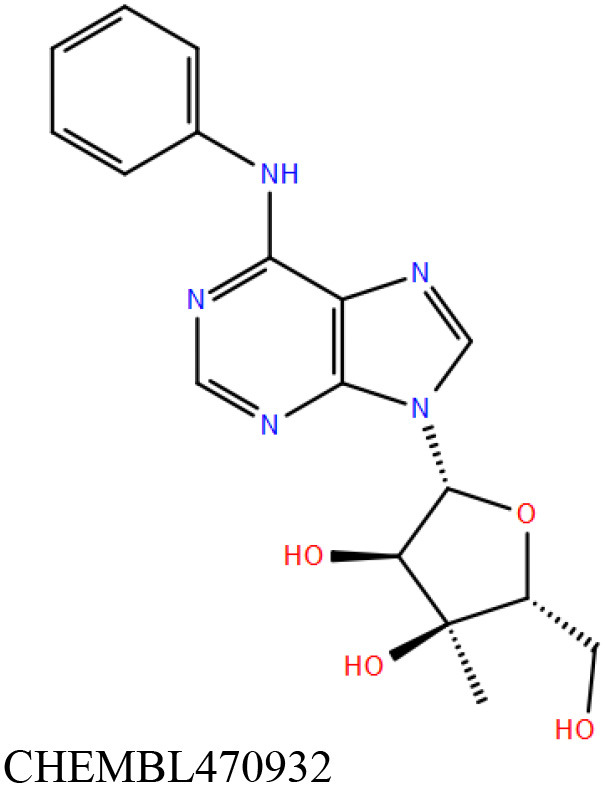	−11.0	−67.4	V9, **G10**, T11, D12, S14, S17, A20, V21, A38, T39, **A40**, Y41, F42, E57, K60, M61, A62, A94, P95, L99, V112, V113, **G114**, N115, **V116**, G117, L118, S128, V129, P130, T146
7	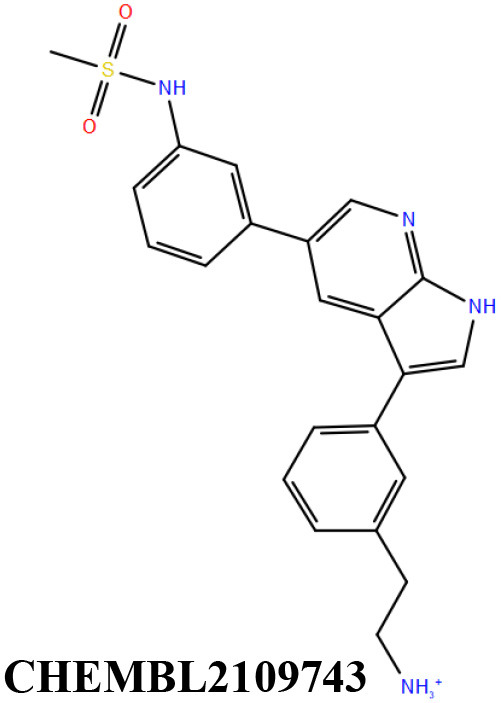	−10.9	−61.6	G10, T11, D12, S16, S17, A38, T39, A40, Y41, F42, **E57**, G58, K60, M61, A62, I67, A94, P95, L99, V113, G114, N115, **V116**, G117, S128, V129, P130, T146
8	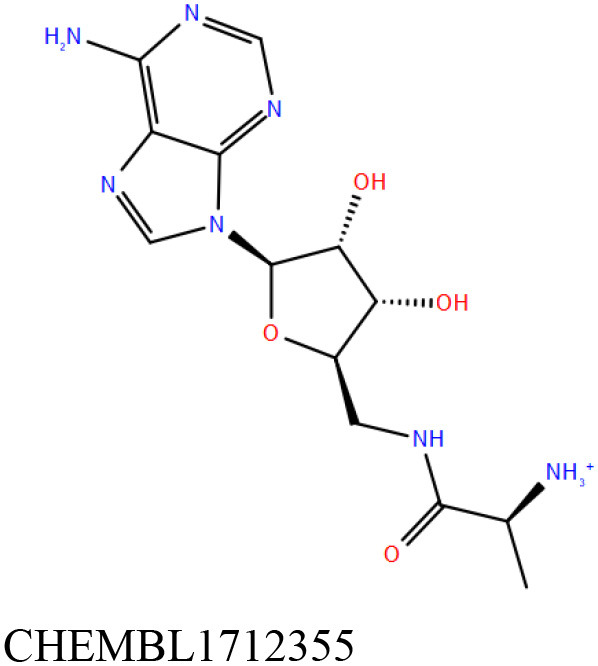	−10.8	−59.4	V9, **G10**, T11, **D12**, S14, **S16**, S17, A20, A38, T39, **A40**, Y41, M61, A62, P95, L99, V112, V113, **G114**, N115, V116, V129, P130, V133, T146
9	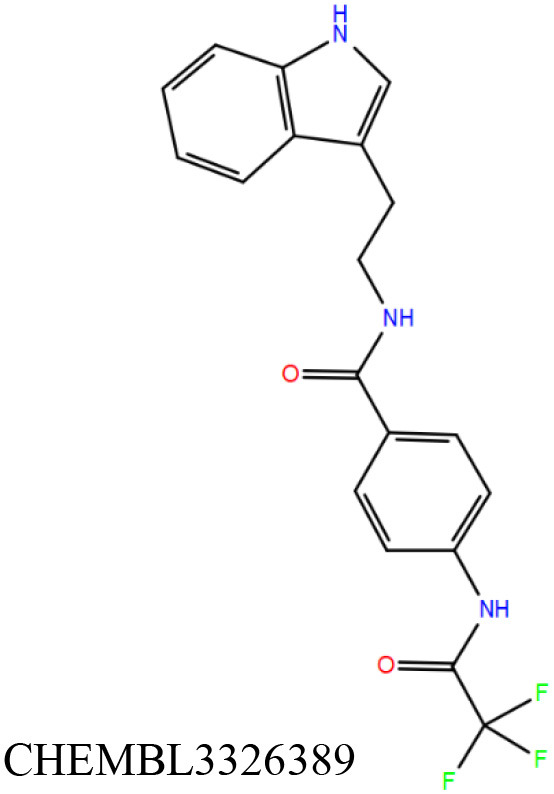	−10.7	−47.6	G10, T11, D12, G13, S14, S16, S17, A20, A38, T39, **A40**, Y41, F42, E57, K60, M61, A62, I67, G93, A94, P95, L99, V113, G114, N115, **V116**, G117, L118, S128, V129, P130
10	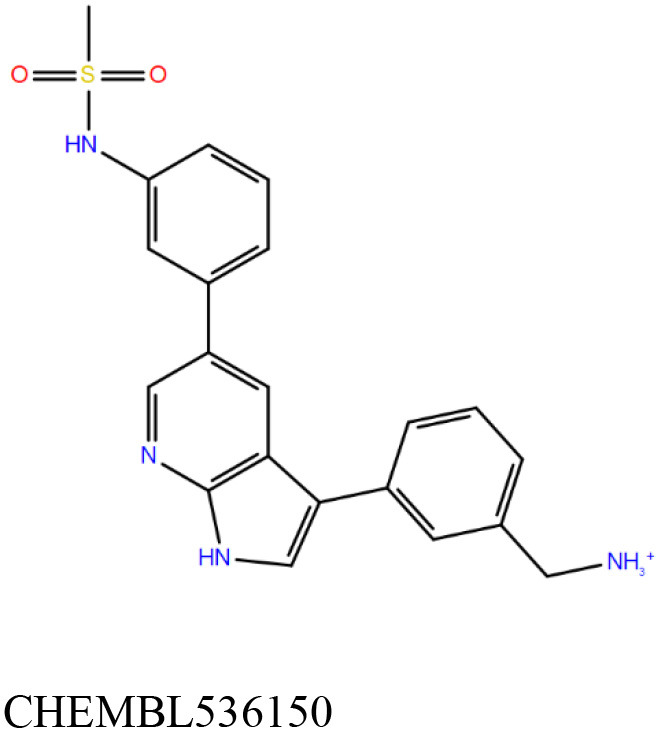	−10.7	−61. 6	G10, T11, D12, S14, S17, A38, T39, A40, Y41, F42, **E57**, K60, M61, A62, A67, G93, A94, P95, L99, V113, G114, N115, **V116**, G117, L128, V129, P130, T146
11	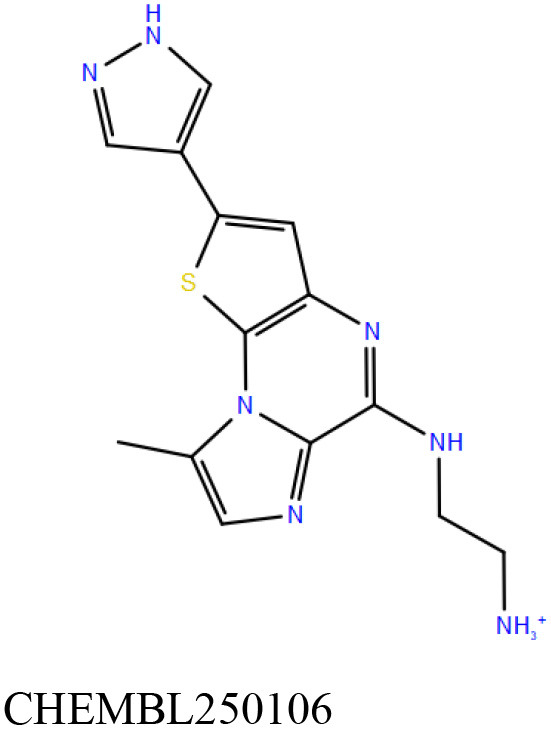	−10.7	−52.5	G10, T11, D12, S17, A38, T39, **A40**, Y41, F42, **E57**, K60, M61, A67, G93, A94, P95, L99, V113, G114, **V116**, G117, L128, V129, P130
12	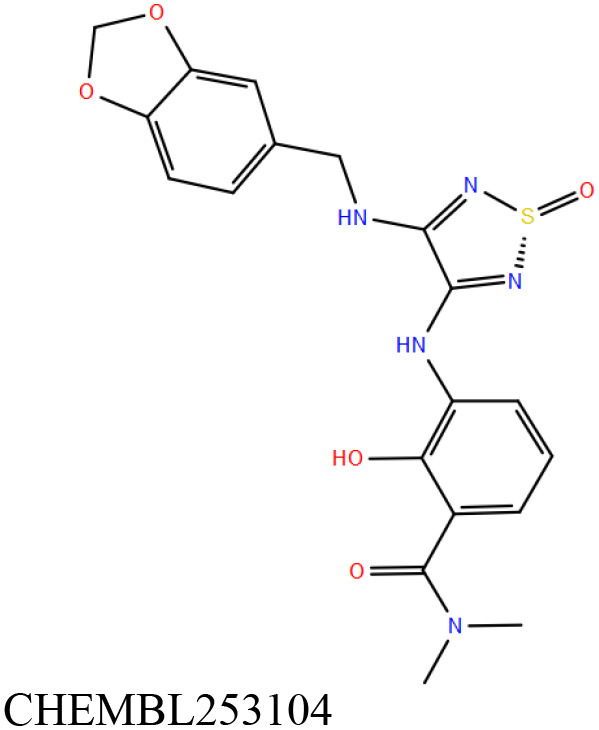	−10.5	−69.6	G10, T11, **D12**, S14, S16, S17, A20, A38, T39, **A40**, M61, A62, V91, P95, A98, L99, V113, **G114**, N115, **V116**, G117, L118, G123, L126, G127, S128, V129, P130, N132, T146
13	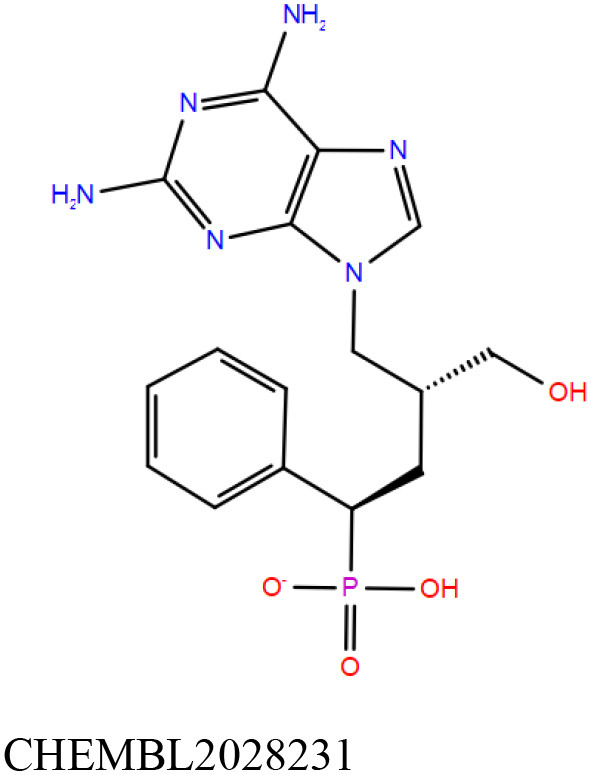	−10.5	−50.8	V9, **G10**, T11, D12, S14, **S16**, S17, A20, V21, A38, T39, **A40**, Y41, M61, A62, V91, P95, L99, V112, V113, **G114**, N115, V116, G117, S128, V129, P130, V133, T146, S147
14	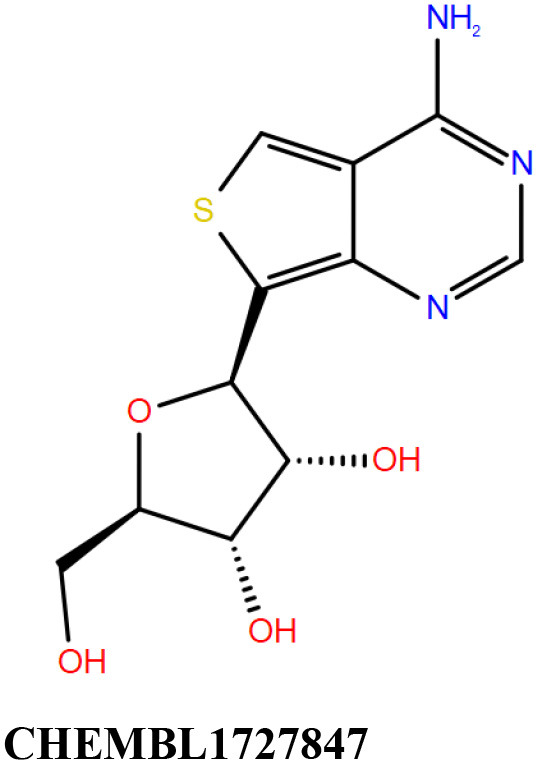	−10.4	−63.7	V9, **G10**, T11, D12, S14, S16, S17, A20, V21, A38, T39, **A40**, Y41, M61, P95, L99, V112, V113, **G114**, N115, **V116**, G117, L118, S128, V129, P130, T146
15	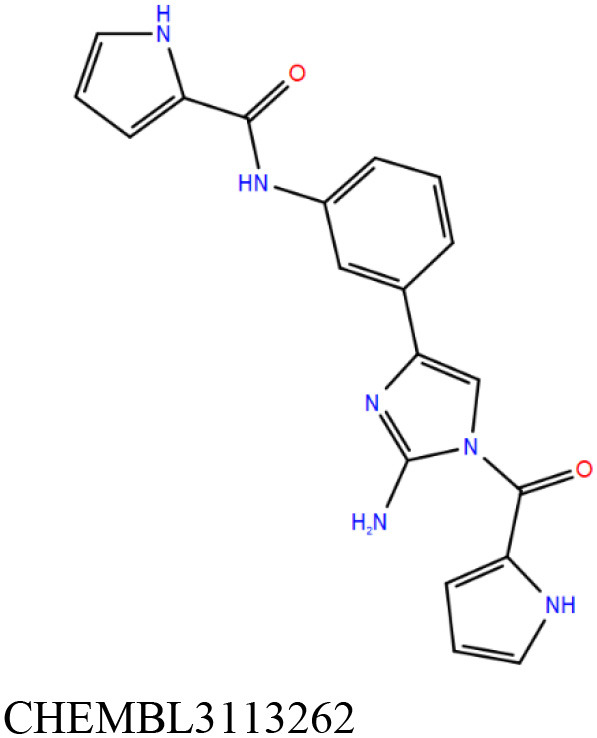	−10.3	−62.0	V9, G10, T11, D12, S17, A20, A38, T39, **A40**, Y41, F42, M61, P95, L99, V113, G114, N115, **V116**, G117, L118, G123, G127, S128, V129, P130, T146
16	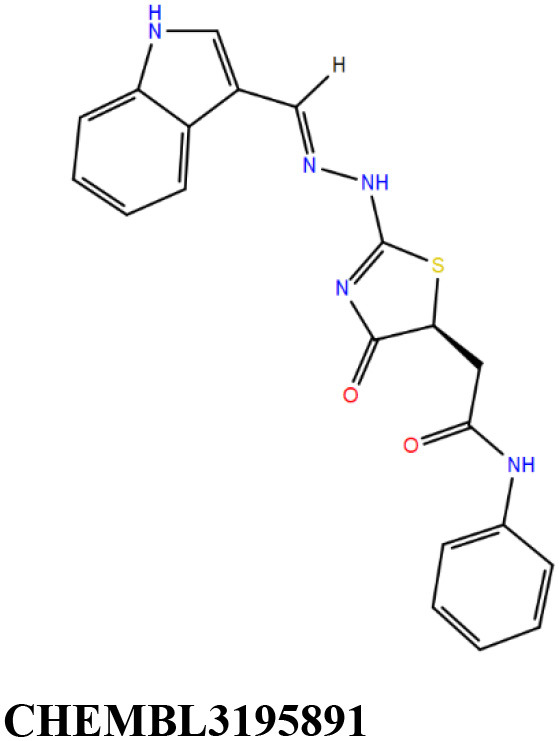	−10.2	−54.3	G10, T11, D12, G13, S14, S17, A38, T39, **A40**, Y41, F42, E57, K60, M61, A62, I67, V91, P95, A98, L99, V113, G114, N115, **V116**, G117, L118, S128, V129, P130
17	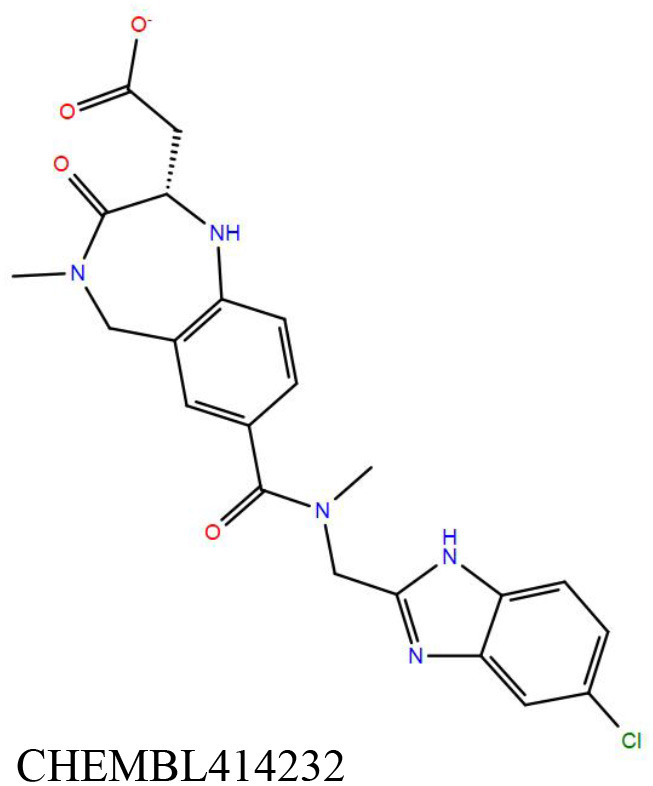	−10.1	−45.0	G10, T11, D12, G13, S14, **S16**, S17, A20, A38, T39, **A40**, Y41, F42, E57, K60, M61, A62, A67, P95, L99, V112, V113, G114, N115, **V116**, G117, S128, V129, P130, V133, **T146**, S147
18	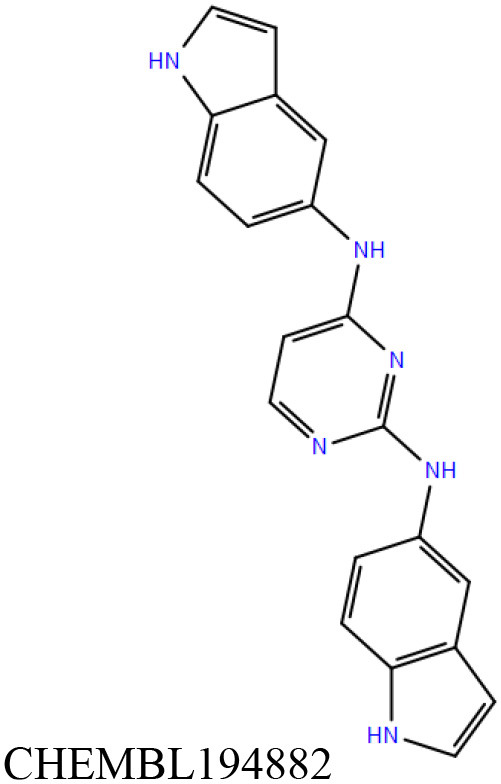	−10.1	−45.0	G10, T11, D12, S17, A38, T39, **A40**, Y41, F42, E57, K60, M61, A62, V91, P95, L99, V113, G114, N115, **V116**, G117, S128, V129, P130
19	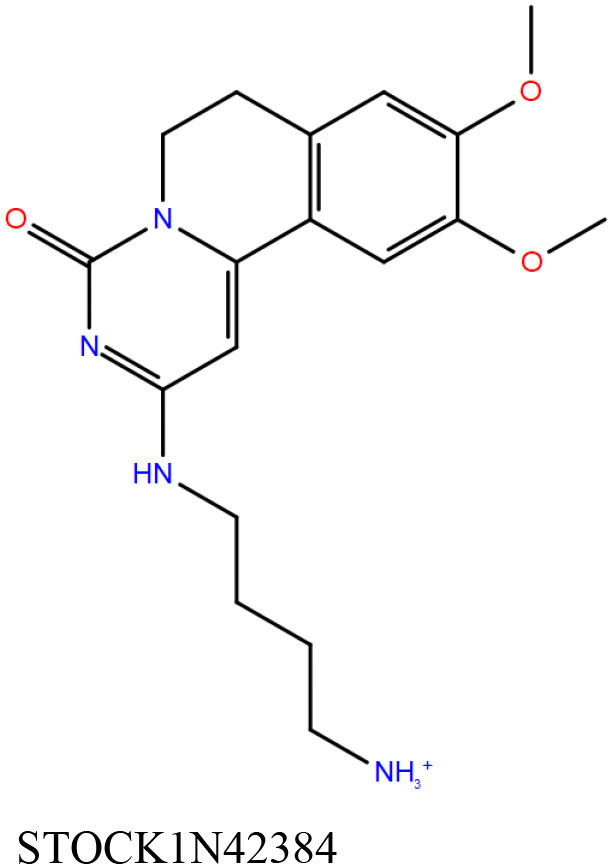	−11.1	−58.2	V9, G10, T11, D12, S14, S16, S17, A38, T39, **A40**, Y41, F42, **E57**, G58, K60, M61, A62, I67, A94, P95, L99, V113, G114, N115, V116, G117, L118, V129, P130, V133, T146
20	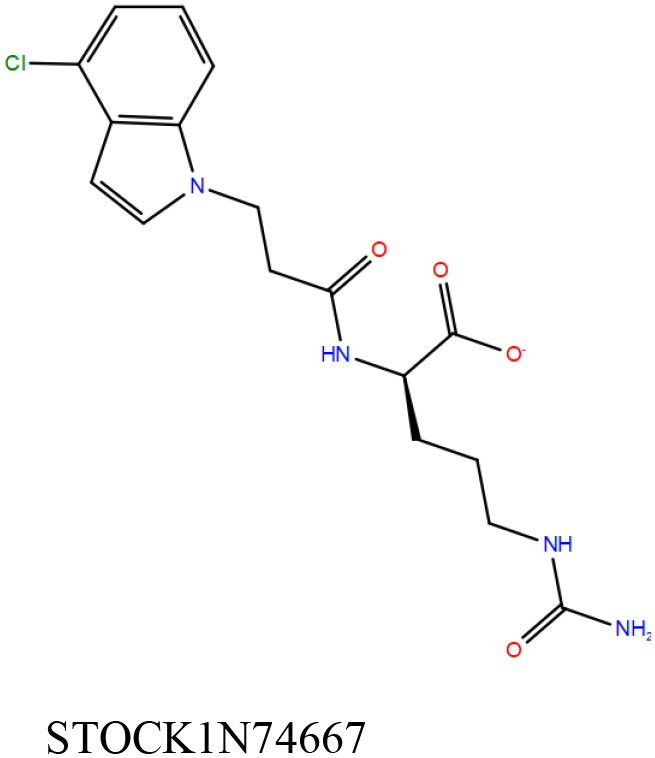	−8.4	−48.1	V9, G10, T11, D12, S14, **S16**, S17, A20, A38, T39, A40, S41, S61, V91, P95, L99, V112, V113, **G114**, N115, **V116**, G117, L118, G123, G127, S128, V129, P130, V133, **T146**, S147
21	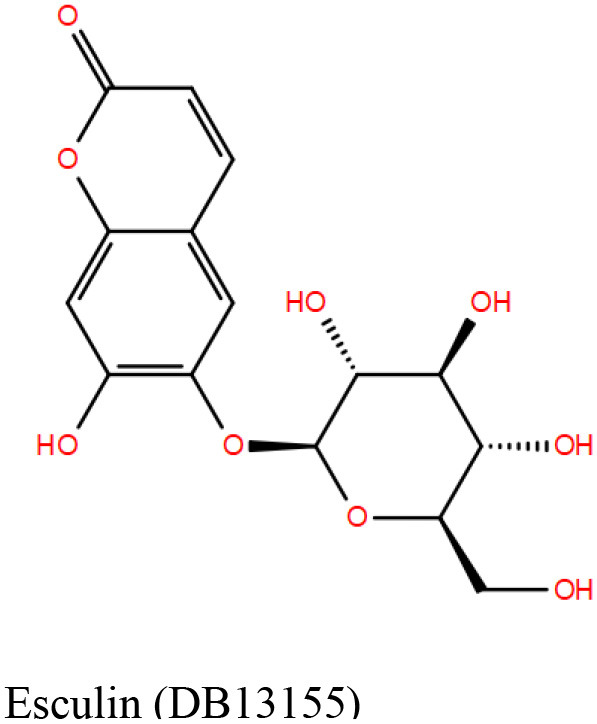	−11.1	−60.4	V9, **G10**, T11, **D12**, S14, S16, S17, A20, A38, T39, **A40**, S61, A62, P95, L99, V112, V113, **G114**, N115, **V116**, G117, L118, S128, **V129**, P130, T146
22	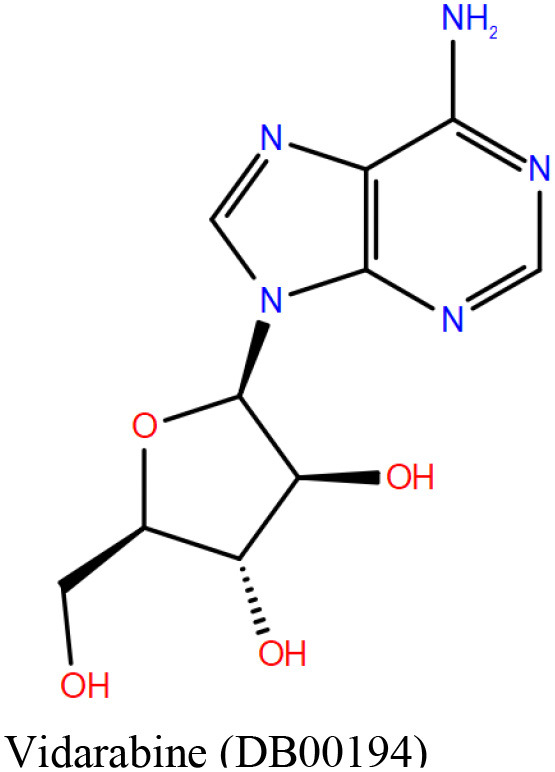	−8.8	−52.6	V9, **G10**, T11, D12, S14, S16, S17, A20, A38, T39, **A40**, S41, M61, P95, L99, V113, G114, N115, **V116**, G117, L118, S128, V129, P130, V133, T146

* *This column holds the information on all binding site residues (within 5 Å) based on the docked pose of the ligand. The residue names in bold are involved in hydrogen bonding. Other residues provide favorable contacts to the ligand. Further details on other types of interaction could be found in Supplementary Table 3*.

*The alphanumeric code indicated below each compound's structure correspond to the original identification number of the compound in the respective databases. The Compound identification numbers represented in bold are the biased set molecules*.

### *In vitro* Analysis

#### Purification of Rv1636 and Microscale Thermophoresis

His-tagged Rv1636 was purified from *E. coli* SP850 *cyc–* strain in a buffer containing 50 mM Tris–Cl (pH 7.5), 100 mM NaCl, 5 mM 2-ME, and 10% glycerol as described earlier (Banerjee et al., 2015). Microscale thermophoresis (MST) was performed on a Nanotemper Technologies Monolith® NT.115 instrument (Munich, Germany). The protein was labeled with NT-495-NHS fluorescent dye in a buffer containing 10 mM HEPES (pH 7.5), 100 mM NaCl, 10% glycerol, and 0.05% Tween20. Labeled His-Rv1636 (100 nM) was added to varying concentrations of the ligand in buffer containing 50 mM Tris–Cl (pH 7.5), 100 mM NaCl, 10% glycerol, and 0.05% Tween20. Samples were incubated at room temperature for 10 min, loaded into capillaries, and placed in the MST block. Thermophoresis was measured at an ambient room temperature of 25°C and performed using 60% excitation power for the nanoblue filter and medium MST IR-laser power. Fluorescent migration used to determine K_d_ was measured from 1.5 to 2.5 s and then normalized to initial fluorescence (−1.0 to 0 s). The data from three independent replicates were analyzed using MO Affinity Analysis software v2.3 and fit to the standard K_d_ fit model, which describes a molecular interaction with a 1:1 stoichiometry according to the law of mass action.

## Results

### Control Library

This library consists of the two known binders of Rv1636 and MSMEG_3811, *viz*., cAMP and ATP. The redocking experiment yielded a reproducible binding pose for cAMP as observed in 5AHW.The two poses (experimental and predicted) perfectly superimpose on each other (Supplementary Figure 3), thus validating our docking protocol. The docked pose of cAMP has a docking score of −10.5 kcal/mol. The docking score of ATP against MSMEG_3811 is −1.7 kcal/mol. This result corroborates the earlier experimental observations (Banerjee et al., 2015). In the current study, too, the binding affinity of cAMP to Rv1636 has been verified through MST assay (Table 2 and Supplementary Figure 4).

**Table 2:** Binding affinity (K_d_) of experimentally tested compounds as determined by MST assays.

**Sl. No**.	**Name of the compound**	**Library**	**Docking score (kcal/mol)**	**Prime-MMGBSA dG_**bind**_ score (kcal/mol)**	**K_**d**_ (μM)**
1	cAMP	Control (positive)	−10.5	−60.5	2.68 ± 0.07
2	STOCK1N42384	Primary (InterBioScreen-natural compound)	−11.1	−58.2	998 ± 82
3	STOCK1N74667	Primary (InterBioScreen-natural compound)	−8.4	−48.1	1717 ± 731
4	Curcumin	Secondary (literature search)	−6.6	−58.8	17.37 ± 0.8

### Primary Library

Compounds from three different sources (ChEMBL, InterBioScreen-natural, and DrugBank-approved) have been collated in this library, as mentioned earlier. Docking results of selected compounds from these libraries are presented below.

#### ChEMBL Library

The initial set of ~2,000 compounds obtained from screening the ChEMBL library yielded compounds whose docking score range from −11.8 to −8.2 kcal/mol. The top 100 compounds (range of docking scores, −11.8 to −10.1 kcal/mol) were subjected to in-depth analysis (Supplementary Table 3). The Prime-MMGBSA dG_bind_ scores of these 100 compounds range from −82.2 to −29.8 kcal/mol. As mentioned earlier, the absolute values calculated here might not agree with experimental binding energies (for details on relevance of Prime-MMGBSA dG_bind_ scores, refer to Materials and Methods). The docking and Prime-MMGBSA dG_bind_ scores calculated in our study indicate favorable binding of the top 100 compounds. Some of these compounds are theoretically better binders than cAMP (as indicated by the scores). Analyzing the interaction profiles of top 100 compounds revealed that most of these docked compounds are engaged in hydrogen bonding with multiple critical binding site residues (like Gly10, Ala40, Ser16, Gly114, etc.). Some of the compounds are also involved in other types of electrostatic interactions (such as salt bridges, aromatic CH–π interactions, π-π stacking, and halogen bonds) with Thr11, Asp12, Phe42, Asp57, etc. Information on the known anti-tubercular property could be obtained from database search for 3 out of the 100 top compounds. These three compounds comprise the “biased” subset of molecules that were shortlisted for experimental investigations. From the remaining 97 compounds, 18 chemically diverse compounds were prioritized for testing that formed the “blind” subset (Table 1). The analyses of the docked poses of two selected *in silico* hits from the ChEMBL library are furnished below.

**Figure 3:**
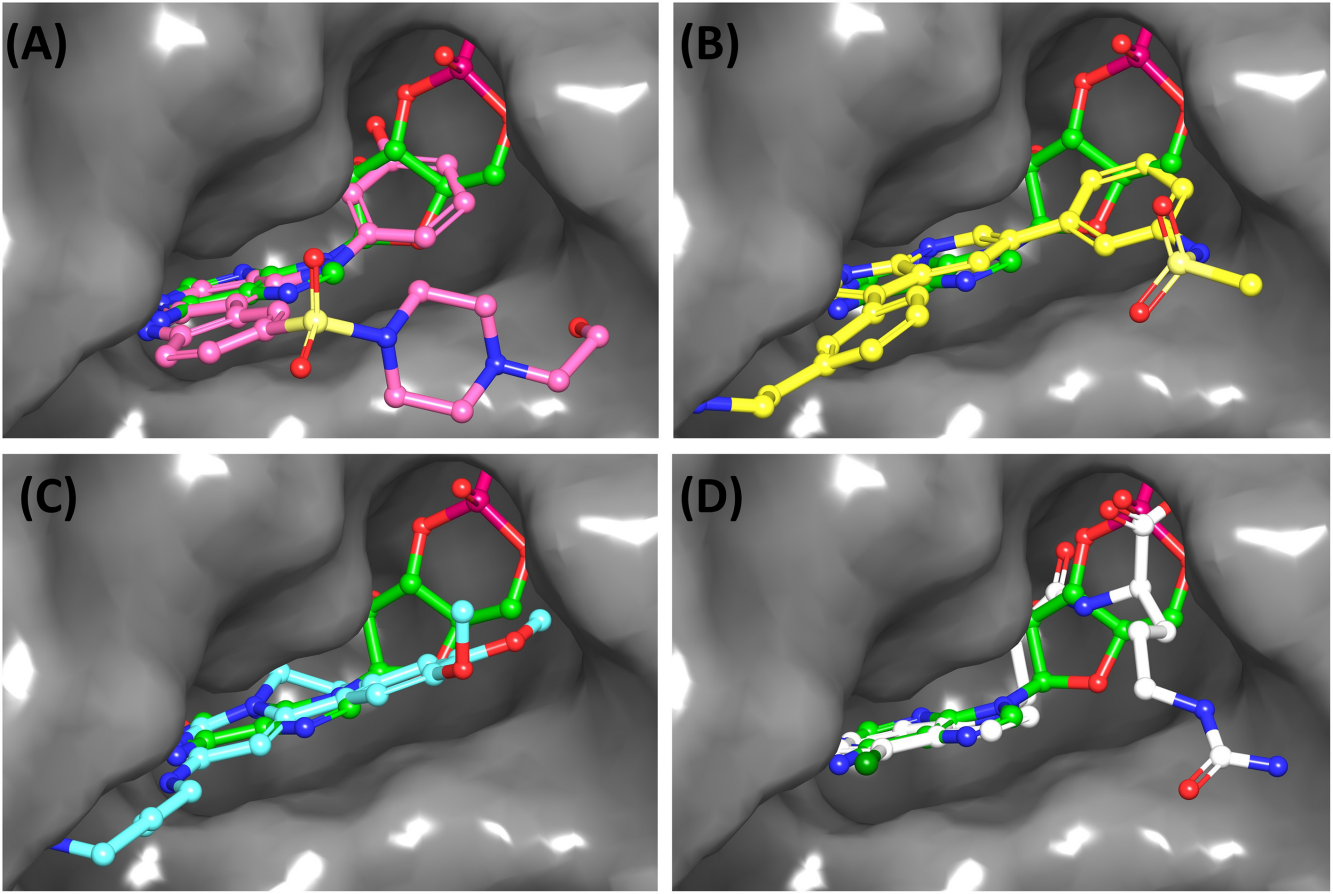
Overlay of docked pose of selected compounds on to the bound pose of cAMP (green stick) as reported in 5AHW. The protein binding site is depicted as gray surface, and the ligands are shown in ball and stick representation: **(A)** ChEMBL3133832 (pink carbon), **(B)** ChEMBL2109743 (yellow carbon), **(C)** STOCK1N-42384 (cyan carbon), **(D)** STOCK1N-74667 (white carbon). Nitrogen, oxygen, chlorine, and sulfur atoms are shown in blue, red, dark green, and yellow, respectively. Hydrogen atoms were not displayed during image generation to maintain visual clarity.

- ChEMBL3133832 (IUPAC name: 3-[[6-[4-(2-hydroxyethyl)piperazin-1-yl]sulfonyl-9*H*-pyrido[2,3-b]indol-4-yl]amino]phenol): This compound is one of the best hits from the ChEMBL library with the best docking score (−11.8 kcal/mol) among the ~0.9 million compounds that were subjected to docking. It also has the best MMGBSA score (−77.1 kcal/mol) among all the compounds that have been selected for testing (Table 1). The compound is well-accommodated in the cAMP binding cavity of MSMEG_3811 and is predicted to be hydrogen-bonded with key residues, such as Gly10, Ala40, Gly114, Val116, and Val129 (Figure 3 and Supplementary Figure 5).
- ChEMBL2109743 (IUPAC name: *N*-[3-[3-[3-(2-aminoethyl)phenyl]-1*H*-pyrrolo[2,3-b]pyridin-5-yl]phenyl]methanesulfonamide): This compound (also referred as GSK581005A) is from the biased subset. Phenotypic screening at GlaxoSmithKline (GSK) led to the identification of ChEMBL2109743/GSK581005A to be effective against *Mtb* H37Rv [minimum inhibitory concentration (MIC) <10 μM] with low human cell-line toxicity (Ballell et al., 2013). Owing to the known antitubercular property of ChEMBL2109743, we have selected it for testing. The docked pose of the compound in cAMP binding site of MSMEG_3811 indicates that it is well-accommodated in the binding pocket (docking score = −10.9 kcal/mol; MMGBSA dG_bind_ score = −61.6 kcal/mol) and is also predicted to be hydrogen bonded with critical binding site residues (Figure 3, Supplementary Figure 6, Table 1, and Supplementary Table 3).

#### InterBioScreen-Natural Library

The 50 initial hits reported from the hierarchical docking exercises have docking scores that range from −11.6 to −7.3 kcal/mol. The relative binding energies of all these 50 compounds were calculated using the Prime-MMGBSA approach. Visual inspection of the poses of all the compounds revealed that only a few of the compounds from this library are engaged in hydrogen bonding with the important binding site residues. Only one compound, STOCK1N-42384 (IUPAC name: 2-((4-aminobutyl)amino)-9,10-dimethoxy-6,7-dihydro-4H-pyrimido[6,1-a]isoquinolin-4-one), has a docking score better than cAMP. Given the importance of natural compounds and their analogs in medicinal chemistry (Mushtaq et al., 2018; Atanasov et al., 2021), two compounds, STOCK1N-42384 and STOCK1N-74667 (IUPAC name: (R)-2-(3-(4-chloro-1H-indol-1-yl)propanamido)-5-ureidopentanoic acid), were prioritized for laboratory testing (Table 1). STOCK1N-42384 and STOCK1N-74667 demonstrated a K_d_ of ~1 and ~1.7 mM, respectively, against Rv1636 (Table 2 and Supplementary Figure 4). The analyses of the docked poses of the two selected compounds from the InterBioScreen-natural library are presented below.

- STOCK1N-42384: This compound engages one of the key binding site residues, Ala40, in hydrogen bonding (Figure 3 and Supplementary Figure 7). Additionally, it is also hydrogen bonded with Glu57. Several binding site residues offer favorable contacts to this compound in its predicted pose, thus contributing to a docking score (−11.1 kcal/mol) comparable to some of the top-scoring virtual hits from the ChEMBL library (Table 1, Figure 3, and Supplementary Figure 7).
- STOCK1N-74667: This compound has a docking score of −8.4 kcal/mol and is predicted to be engaged in hydrogen bonding with Gly114, one of the critical residues for cAMP binding as demonstrated in our previous mutagenesis studies and discussed earlier. It is also predicted to be hydrogen bonded to Ser16, Val116, and Thr146. Furthermore, several residues housed by the cAMP binding site offer favorable contacts to the docked pose of STOCK1N-74667 in MSMEG_3811 (Table 1, Figure 3, and Supplementary Figure 8).

#### DrugBank Library

A list of 20 initial reported hits were obtained upon screening the library of approved drugs against both the protein conformers. The docked poses of all the hits were analyzed, and two compounds were prioritized for experimental testing: esculin and vidarabine (Table 1). While esculin (https://go.drugbank.com/drugs/DB13155) is a glycosyl compound used as a vasoprotective agent, vidarabine is a known antiviral drug with established safety records (https://www.drugbank.ca/drugs/DB00194). The primary target of esculin is a human protein (androgen receptor). In addition, esculin is a coumarin derivative, and promiscuity of this chemical class of compounds is well-known (Stefanachi et al., 2018). Therefore, anticipating esculin could interfere with the functions of undesired human proteins, vidarabine was assigned a higher priority for further investigations. While docking studies predicted favorable interactions of vidarabine and esculin with critical binding site residues (Table 1), *in vitro* binding assays performed on both these compounds against Rv1636 did not show encouraging results (data not shown).

### Secondary Library

The 10 polyphenolic natural compounds from this library (that includes curcumin) have docking scores between −7.3 and −4.8 kcal/mol, which indicate that the binding affinities of these polyphenolic compounds are likely to be weaker than the native ligand, cAMP (Supplementary Table 4). MST assays revealed a K_d_ of 17 μM for curcumin against Rv1636 (Table 2 and Supplementary Figures 4, 9). The two other approved drugs from the secondary library, amikacin and kanamycin, although predicted to be favorably accommodated (−9.5 and −8.9 kcal/mol, respectively) in Rv1636, have docking scores poorer than most of the compounds that we have selected for experimental validations. The majority of the compounds in the secondary library failed to establish hydrogen bonds with the critical binding site residues (Supplementary Table 4).

## Discussion

This study has shortlisted potential binders of *Mtb* USP (Rv1636) using a rigorous computational protocol primarily employing molecular docking simulations. Although docking scores are important parameters to find “needles” (potential binders) from the “haystack” (of non-binders in a large chemical universe), it is well-known that the scoring functions can have their own limitations (Huang et al., 2010). Therefore, to derive meaningful insights from computational studies, we have integrated other physics-based methods like the Prime-MMGBSA calculations and the available experimental knowledge in our workflow to ensure that the high confidence virtual hits are taken forward for experimental validations. A molecule with more negative docking and Prime-MMGBSA dG_bind_ scores and also predicted to be hydrogen bonded with the critical binding site residues (as shown previously through mutagenesis experiments) are expected to be better candidates. Furthermore, when such a candidate is also reported as hits against both conformers I and II, the confidence associated with that compound is higher as explained earlier (Supplementary Table 3). It is to be noted that the ChEMBL and InterBioScreen-Natural compound libraries of molecules have been filtered through REOS, PAINS, and Lipinski's rule of five filters as a prescreening step. Therefore, all the shortlisted candidates from these libraries are drug-like molecules and, in general, can be assumed to be safe. The targeted pocket in Rv1636 is suited for binding nucleotide scaffolds. Therefore, any molecule with a nucleotide containing moiety or its analog targeting this pocket has a chance to cross-talk with host nucleotide-binding proteins, such as the protein kinases (Taylor et al., 2012). Nevertheless, ChEMBL2109743, while known to inhibit human serum and glucocorticoid-regulated kinase 1 (SGK-1) (James et al., 2009), has been observed to have low cellular toxicity in the GSK tuberculosis screening.

### Corroboration Between Our *in silico* Studies and Experimental Findings

#### cAMP and ATP (Control Library)

As mentioned earlier, cAMP binds to the conserved USP domain of Rv1636 by engaging some key binding site residues in hydrogen bonding confirmed through structure-guided mutagenesis studies. Furthermore, cAMP has been found to bind to this protein with almost 10 times higher affinity than ATP (Banerjee et al., 2015). Similar observations have also been noted in our computational studies, as discussed earlier (Supplementary Figure 3 and Supplementary Table 3).

#### Biased Subset (ChEMBL Library)

ChEMBL2109743/GSK581005A is one of the interesting hits that we identified by screening the ChEMBL library of compounds. In a study by Mugumbate et al., it has been suggested through chemogenomic studies that *Mtb* dihyrofolate reductase (DHFR) could be a target of this compound (Mugumbate et al., 2015). Our virtual screening results suggest that GSK581005A is a potential binder of Rv1636 and is predicted to be hydrogen-bonded with critical binding site residues (Figure 3, Supplementary Figure 6, Table 1, and Supplementary Table 3). It is worthy to note that chemogenomic tools used in the mentioned study were trained on the ChEMBL database of known target–ligand pairs. Therefore, all predicted targets are biased toward well-studied proteins that are already included to the ChEMBL database (Mugumbate et al., 2015). Hence, it is unlikely that such methods would predict Rv1636 as a target of any query compound. Given that GSK581005A is already privileged with respect to its cell permeability, there is merit in future *in vitro* testing of this compound against Rv1636 (Manjunatha and Smith, 2015).

Two other compounds (ChEMBL3195891 and ChEMBL17272847) have been reported to be tested against an *Mtb* target. Both these compounds were reported to be active in a counter-screening for inhibitors of the fructose-bisphosphate aldolase (FBA) (https://pubchem.ncbi.nlm.n ih.gov/compound/135470622#section=Biological-Test-Results). However, a detailed report that can aid in making the informed decisions could not be found.

#### STOCK1N-42384 and STOCK1N-74667 (InterBioScreen-Natural Library)

Both these natural compounds shortlisted from our virtual screening bound Rv1636 in the MST assays (Table 2 and Supplementary Figure 4). Further investigations with analogs of these compounds that establish hydrogen bonds with the critical cAMP binding residues (such as Gly 10 and Gly114 for STOCK1N-42384; Gly10 and Ala40 for STOCK1N-74667) could improve the binding affinities of these natural compounds (Table 1, Figure 3, and Supplementary Figures 5, 6).

#### Curcumin (Secondary Library)

A previously published report (Aanandhi et al., 2014) indicated curcumin to be a potential binder of Rv1636 at a site different from the cAMP binding site. Analysis of the docking score and hydrogen-bonding profile of curcumin at cAMP binding site in our study hints that curcumin is unlikely to be a strong binder (Supplementary Table 4 and Supplementary Figure 9). Contrary to our expectation, curcumin demonstrated a high binding affinity toward Rv1636 (K_d_ ~17 μM) (Table 2 and Supplementary Figure 4). Therefore, curcumin may interact with Rv1636 through a site different from the cAMP binding site. Notably, curcumin and other polyphenolic compounds are highly promiscuous and are flagged as pan-assay interference compounds (PAINS) that often act by non-drug-like mechanisms. Attempts to optimize PAINS as drug candidates in the past have proven futile, and we do not encourage medicinal chemists to consider curcumin as a good starting point for designing an inhibitor against Rv1636 or any other drug targets (Baell, 2010; Baell and Holloway, 2010; Baell and Walters, 2014).

A close inspection of the cAMP binding site in 5AHW reveals that the site can be grossly divided into five subpockets (P1, P2, P3, P4, and P5) (Supplementary Figure 10). The P1 comprises a mixture of hydrophobic and polar residues (Gly10, Thr11, Asp12, Ser14, Val113, Gly114, Asn115, and Val116). The P2 (Ala38, Thr39, Ala40, and Leu99), P3 (Met61 and Pro95), and P4 (Ala20, Val129, Pro130) are predominantly hydrophobic. Interestingly, the backbones of some of the hydrophobic residues in P1 and P2 are directed toward the ligand-binding cavity, thus facilitating the formation of hydrogen bonds with cAMP. The residues in P3 and P4 offer favorable hydrophobic contacts to the bound cAMP. The P5 is formed by three polar residues, *viz*., Ser16, Ser17, and Thr146. Ser16 and Thr146 are involved in side-chain-mediated hydrogen-bonding interactions with bound cAMP in 5AHW (Supplementary Figure 10). Analysis of the docked poses of the top 100 compounds shows that most of the compounds in our library do not establish hydrogen bonds with the P5 residues (Supplementary Table 3). Introduction of functional groups at appropriate sites on the ligand that help form hydrogen bonds with P5 residues may contribute to improving the binding affinities of the compounds against Rv1636. In the future, such chemical modifications of the shortlisted compounds that show promising activity in experimental testing can be explored. Furthermore, molecular dynamics studies on promising *in vitro* hits would provide important insights into compound optimization.

The *in silico*-driven approach employed in this study has helped in shortlisting 22 virtual hits that can potentially bind to *Mtb* USP (Rv1636). This is the first report of screening a large library of publicly available compounds (~1.9 million) to identify potential binders of Rv1636. An overall analysis of the results on the shortlisted candidates suggests that these compounds are likely to be better candidate binders of cAMP binding site in Rv1636 than earlier reported polyphenolic compounds. *In vitro* testing of a few shortlisted compounds has demonstrated promising candidates from the natural compound library. Corroboration between our computational and experimental studies emphasizes the strength of a carefully designed protocol used in selecting an enriched set of potential compounds from vast chemical libraries. Relevant details of the shortlisted compounds that might help medicinal chemists, biochemists, and other researchers make an informed decision for selecting, testing, and designing experiment protocols have been provided (Tables 1, 2 and Supplementary Tables 3, 5). To conclude, the findings reported in this study can serve as important starting points in the drug discovery pipeline of antitubercular leads targeted against Rv1636. It can be expected that successful inhibition of this protein combined with other established anti-tubercular therapeutic approaches could open new avenues for effective disease management and tackling the emerging problem of drug resistance. Finally, the integrative *in silico* pipeline presented in this study is a generalized one and, in principle, can be used for any target-centric ligand screening ventures.

## Data Availability Statement

The original contributions presented in the study are included in the article/Supplementary Material, further inquiries can be directed to the corresponding author/s.

## Author Contributions

SC designed and performed the computational studies. MC and AB performed experiments. SC prepared the manuscript with inputs from all the authors. SC, NS, and SV finalized the manuscript.

## Conflict of Interest

The authors declare that the research was conducted in the absence of any commercial or financial relationships that could be construed as a potential conflict of interest.

## Supplementary Material

Supplementary Figure 1 Structural comparison of apo Rv1636 (PDB code: 1TQ8) and cAMP bound MSMEG_3811 (PDB code: 5AHW). **(A)** Superimposition of chain C of 5AHW (cyan cartoon) on to chain A of 1TQ8 (violet cartoon). The overall RMSD between the aligned residues in these two chains as calculated using DALI (Holm, 2020) is 3.1Å. The residues within 5Å of bound cAMP (depicted in dot representation with green carbon atoms) are shown in magenta in both 5AHW and 1TQ8. The parts of the protein chains that superimpose well are shown as translucent cartoons whereas the parts showing deviations are shown as opaque cartoons. The regions that show structural deviations of the secondary structures are encircled with black dashes. The region encircled with orange dashes could be seen only in 5AHW. The protein residues in the equivalent region are missing the electron density map of 1TQ8. **(B)** For better visualization, only the parts that show deviations are shown; the rest of the parts in the two proteins that superimposes well were not displayed during image generation. **(C)** Superimposition of all the binding site residues surrounding cAMP (represented as sphere with green carbon atoms). **(D)** The pair of residues which show structural deviations are shown (Y41:Y53; P95:P107; L99:L111; N115:N127; V129:V141; P130:P142; V133:V145; T146:T158; S147:T159). The co-ordinates of the residue equivalent to M61 (of 5AHW) are missing in the electron density map of 1TQ8. In **(C,D)** protein residues are shown as thin sticks; 1TQ8: violet, 5AHW: magenta. The residue identifiers are also color-coded as per the color of the corresponding residues.

Supplementary Figure 2 Analysis of crystal structure of cAMP bound MSMEG_3811 (PDB code: 5AHW). **(A)** The six chains of MSMEG_3811 are shown in surface representation (chain A: green, chain B: blue, chain C: cyan, chain D: yellow, chain E: wheatish, chain F: white). The binding site of cAMP (represented as dots; green carbon atoms) in chain C is shown in magenta (also indicated by a black arrow). As can be seen in the figure, the magenta region only spans within the chain C and is away from the interface of the protomers. **(B)** cAMP in the binding site (shown as magenta translucent surface) of chain C of 5AHW. The residue V113 in the binding site is shown as gray stick. The atoms CA, CB, CG1, and CG2 of V113 have dual occupancies and thus two different orientations of the side chain could be seen. **(C)** The side-chain orientation of conformer I of V113 with respect to bound cAMP in chain C. **(D)** The side-chain orientation of conformer II of V113 with respect to bound cAMP in chain C. cAMP is shown in stick representation in panel B, C, and D with green carbon atoms. Nitrogen, oxygen, and phosphorous atoms are shown in blue, red and orange, respectively.

Supplementary Figure 3 cAMP in MSMEG_3811 binding pocket (PDB code: 5AHW). **(A)** Superimposition of experimentally determined bound pose of cAMP (green) on to the re-docked pose of cAMP (yellow) in the binding pocket (gray surface) of the protein. **(B)** cAMP (green ball and stick model with gray transparent surface) bound to the protein binding site. The residues (within 5Å of the ligand) in the binding pocket are shown as thin sticks and color-coded based on their physicochemical properties (cyan: polar; green: hydrophobic; red: charged and negative; white: glycine). **(C)** 2D-interaction diagram of cAMP with residues in the binding pocket. Hydrogen bonds are shown in pink arrows. The residue identifiers are depicted as leaves, where the base of the leaves indicate the residue backbone, and the tip of the leaves indicate the direction in which the side-chains of the residues are pointed. Nitrogen, oxygen, sulfur, and phosphorus atoms are shown in blue, red, yellow and orange, respectively. Hydrogen atoms were not displayed during image generation to maintain visual clarity.

Supplementary Figure 4 Micro-scale thermophoresis to study interaction of fluorescent tagged-His-Rv1636 (100 nM) with varying concentration of: **(A)** cAMP, **(B)** STOCK1N-42834 and STOCK1N-74667, **(C)** Curcumin.

Supplementary Figure 5 2D interaction map of docked pose of ChEMBL3133832 with the binding site residues of MSMEG_3811. For details related to color code, please refer to the legend to Supplementary Figure 3.

Supplementary Figure 6 2D interaction map of docked pose of ChEMBL2109743 with the binding site residues of MSMEG_3811. For details related to color code, please refer to the legend to Supplementary Figure 3.

Supplementary Figure 7 2D interaction map of docked pose of STOCK1N-42384 with the binding site residues of MSMEG_3811. For details related to color code, please refer to the legend to Supplementary Figure 3.

Supplementary Figure 8 2D interaction map of docked pose of STOCK1N-74667 with the binding site residues of MSMEG_3811. For details related to color code, please refer to the legend to Supplementary Figure 3.

Supplementary Figure 9 Analysis of docked pose of Curcumin with the binding site residues of MSMEG_3811. **(A)** Overlay of docked pose of curcumin (violet stick) onto bound pose of cAMP (green stick). Nitrogen, and oxygen atoms are shown in blue and red, respectively. Hydrogen atoms were not displayed during image generation to maintain visual clarity. **(B)** 2D interaction map of docked pose of curcumin with the binding site residues of MSMEG_3811. For details related to color code, please refer to the legend to Supplementary Figure 3.

Supplementary Figure 10 Sub-pockets in cAMP binding site of MSMEG_3811 (PDB code: 5AHW). The five sub-pockets are marked P1, P2, P3, P4, and P5. The group of residues in each sub-pocket is encircled. For details related to color code, please refer to the legend to Supplementary Figure 3.

Supplementary Table 1 EDIA report for cAMP and the binding site residues in chain C of 5AHW.

Supplementary Table 2 Geometric criteria used for detecting non-covalent interactions between protein and the docked ligands.

Supplementary Table 3 Docking and Prime-MMGBSA results with molecular properties of top 100 compounds screened from the ChEMBL library.

Supplementary Table 4 Docking and Prime-MMGBSA results of compounds from secondary library.

Supplementary Table 5 Chemical similarity (expressed as Tanimoto coefficients) matrix of top 100 compounds from ChEMBL library.
