# Supplementary figures and images for: Identification of Potential Binders of Mtb Universal Stress Protein (Rv1636) Through an in silico Approach and Insights Into Compound Selection for Experimental Validation
Source: Front Mol Biosci. 2021 May 3;8:599221. doi: 10.3389/fmolb.2021.599221 (PMC8126637; doi:10.3389/fmolb.2021.599221)

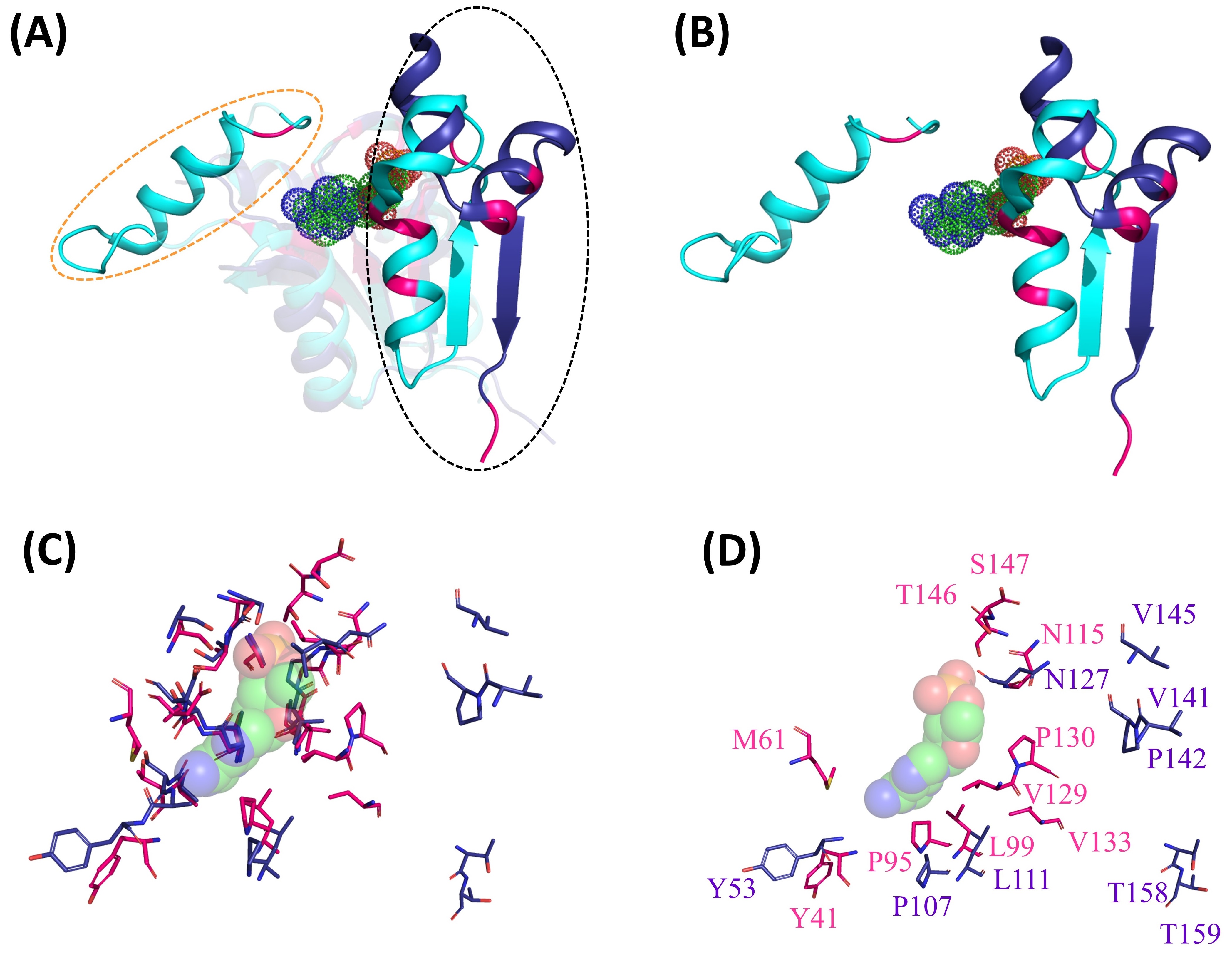

Supplement: Supplementary Figure 1 — Structural comparison of apo Rv1636 (PDB code: 1TQ8) and cAMP bound MSMEG_3811 (PDB code: 5AHW). (A) Superimposition of chain C of 5AHW (cyan cartoon) on to chain A of 1TQ8 (violet cartoon). The overall RMSD between the aligned residues in these two chains as calculated using DALI (Holm, 2020) is 3.1Å. The residues within 5Å of bound cAMP (depicted in dot representation with green carbon atoms) are shown in magenta in both 5AHW and 1TQ8. The parts of the protein chains that superimpose well are shown as translucent cartoons whereas the parts showing deviations are shown as opaque cartoons. The regions that show structural deviations of the secondary structures are encircled with black dashes. The region encircled with orange dashes could be seen only in 5AHW. The protein residues in the equivalent region are missing the electron density map of 1TQ8. (B) For better visualization, only the parts that show deviations are shown; the rest of the parts in the two proteins that superimposes well were not displayed during image generation. (C) Superimposition of all the binding site residues surrounding cAMP (represented as sphere with green carbon atoms). (D) The pair of residues which show structural deviations are shown (Y41:Y53; P95:P107; L99:L111; N115:N127; V129:V141; P130:P142; V133:V145; T146:T158; S147:T159). The co-ordinates of the residue equivalent to M61 (of 5AHW) are missing in the electron density map of 1TQ8. In (C,D) protein residues are shown as thin sticks; 1TQ8: violet, 5AHW: magenta. The residue identifiers are also color-coded as per the color of the corresponding residues. [file Image_1.JPEG]

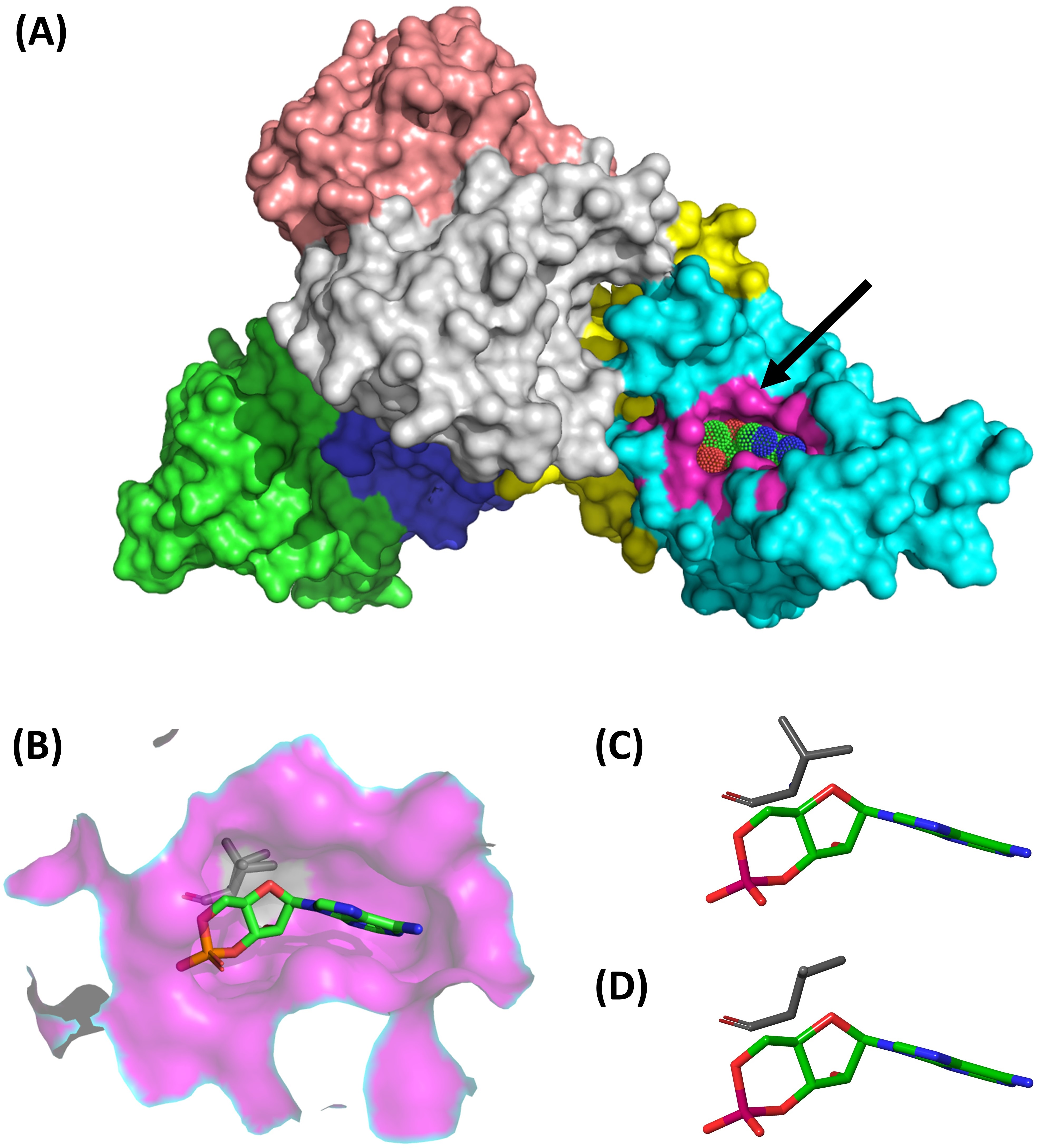

Supplement: Supplementary Figure 2 — Analysis of crystal structure of cAMP bound MSMEG_3811 (PDB code: 5AHW). (A) The six chains of MSMEG_3811 are shown in surface representation (chain A: green, chain B: blue, chain C: cyan, chain D: yellow, chain E: wheatish, chain F: white). The binding site of cAMP (represented as dots; green carbon atoms) in chain C is shown in magenta (also indicated by a black arrow). As can be seen in the figure, the magenta region only spans within the chain C and is away from the interface of the protomers. (B) cAMP in the binding site (shown as magenta translucent surface) of chain C of 5AHW. The residue V113 in the binding site is shown as gray stick. The atoms CA, CB, CG1, and CG2 of V113 have dual occupancies and thus two different orientations of the side chain could be seen. (C) The side-chain orientation of conformer I of V113 with respect to bound cAMP in chain C. (D) The side-chain orientation of conformer II of V113 with respect to bound cAMP in chain C. cAMP is shown in stick representation in panel B, C, and D with green carbon atoms. Nitrogen, oxygen, and phosphorous atoms are shown in blue, red and orange, respectively. [file Image_2.JPEG]

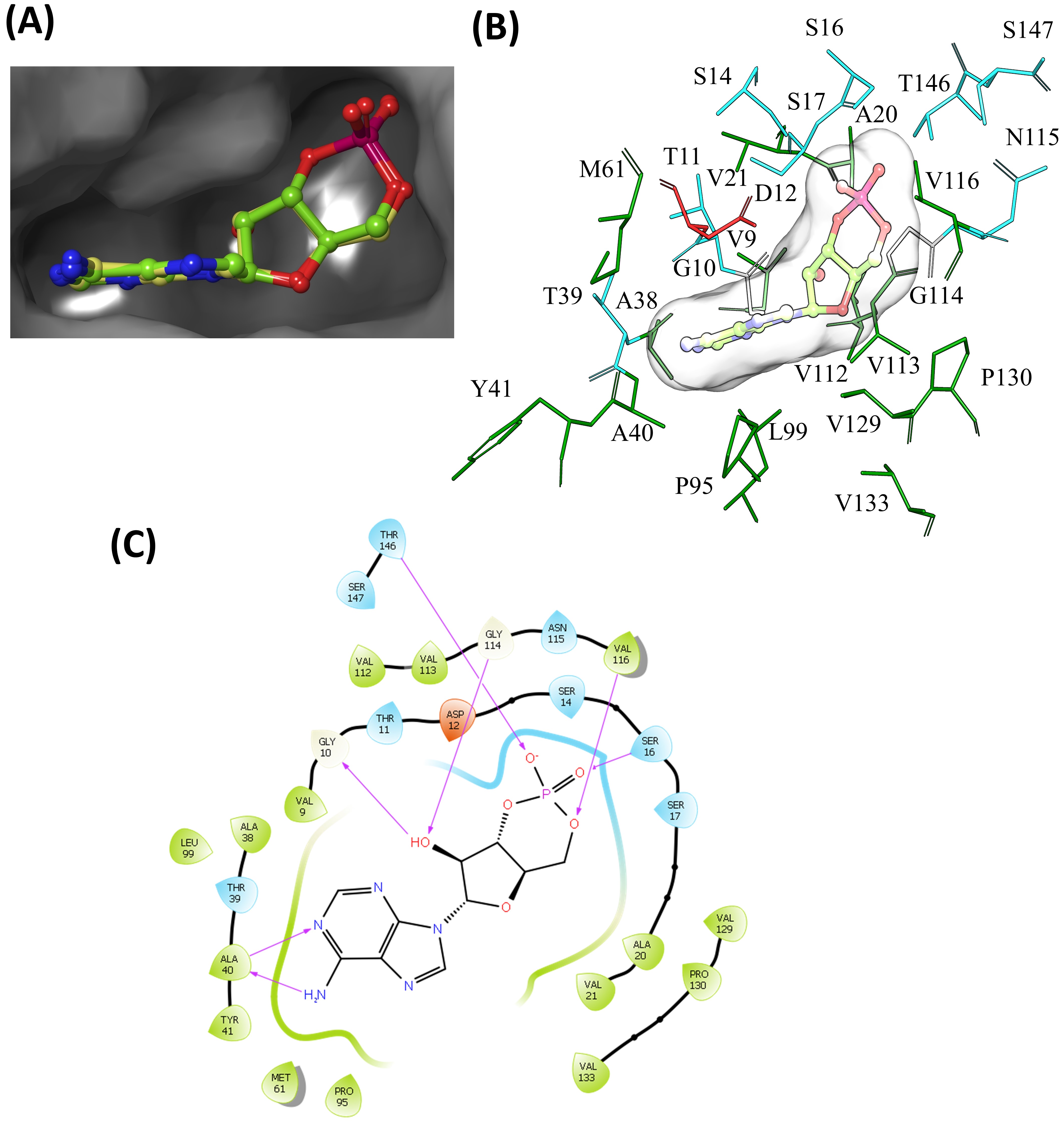

Supplement: Supplementary Figure 3 — cAMP in MSMEG_3811 binding pocket (PDB code: 5AHW). (A) Superimposition of experimentally determined bound pose of cAMP (green) on to the re-docked pose of cAMP (yellow) in the binding pocket (gray surface) of the protein. (B) cAMP (green ball and stick model with gray transparent surface) bound to the protein binding site. The residues (within 5Å of the ligand) in the binding pocket are shown as thin sticks and color-coded based on their physicochemical properties (cyan: polar; green: hydrophobic; red: charged and negative; white: glycine). (C) 2D-interaction diagram of cAMP with residues in the binding pocket. Hydrogen bonds are shown in pink arrows. The residue identifiers are depicted as leaves, where the base of the leaves indicate the residue backbone, and the tip of the leaves indicate the direction in which the side-chains of the residues are pointed. Nitrogen, oxygen, sulfur, and phosphorus atoms are shown in blue, red, yellow and orange, respectively. Hydrogen atoms were not displayed during image generation to maintain visual clarity. [file Image_3.JPEG]

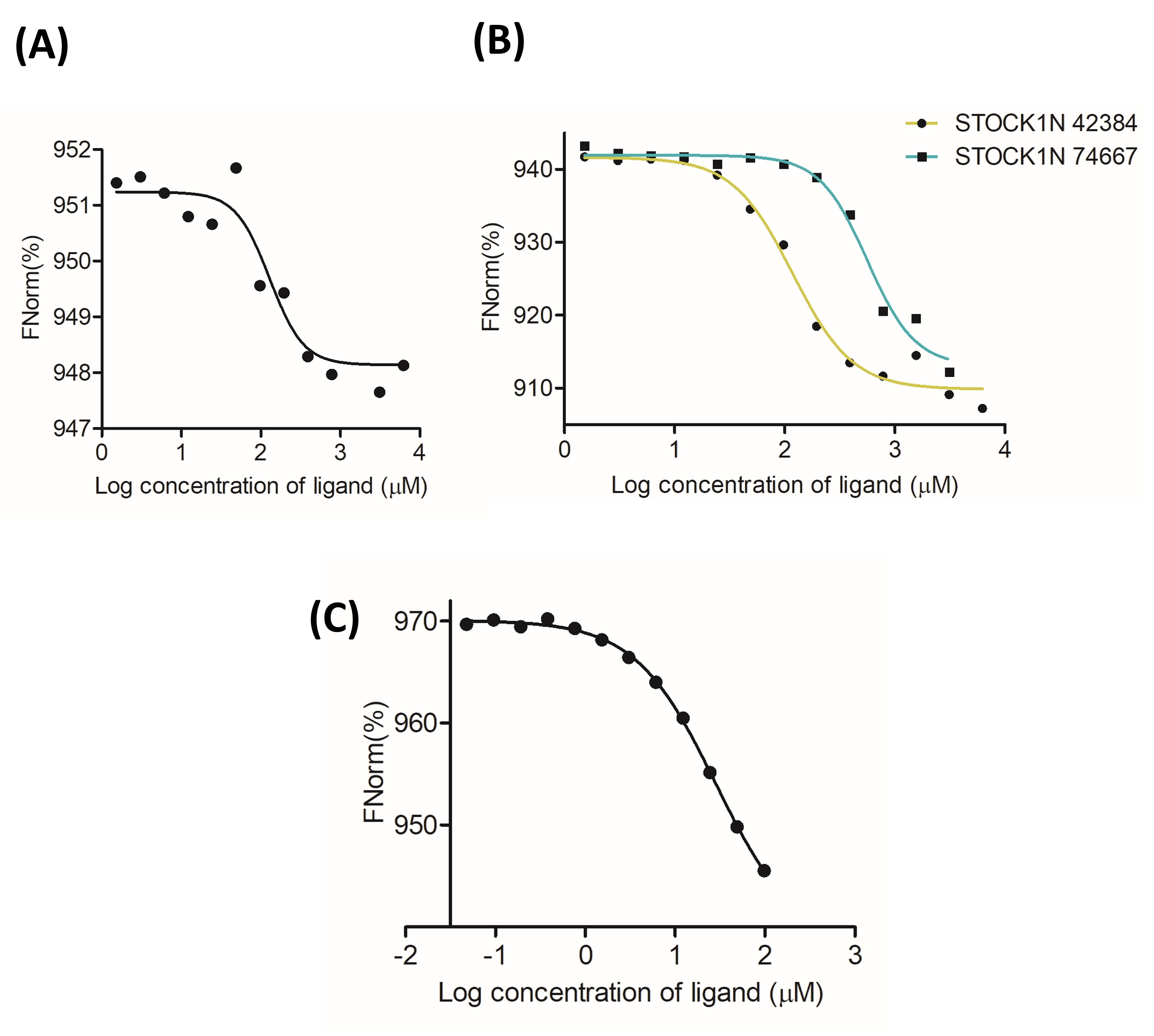

Supplement: Supplementary Figure 4 — Micro-scale thermophoresis to study interaction of fluorescent tagged-His-Rv1636 (100 nM) with varying concentration of: (A) cAMP, (B) STOCK1N-42834 and STOCK1N-74667, (C) Curcumin. [file Image_4.JPEG]

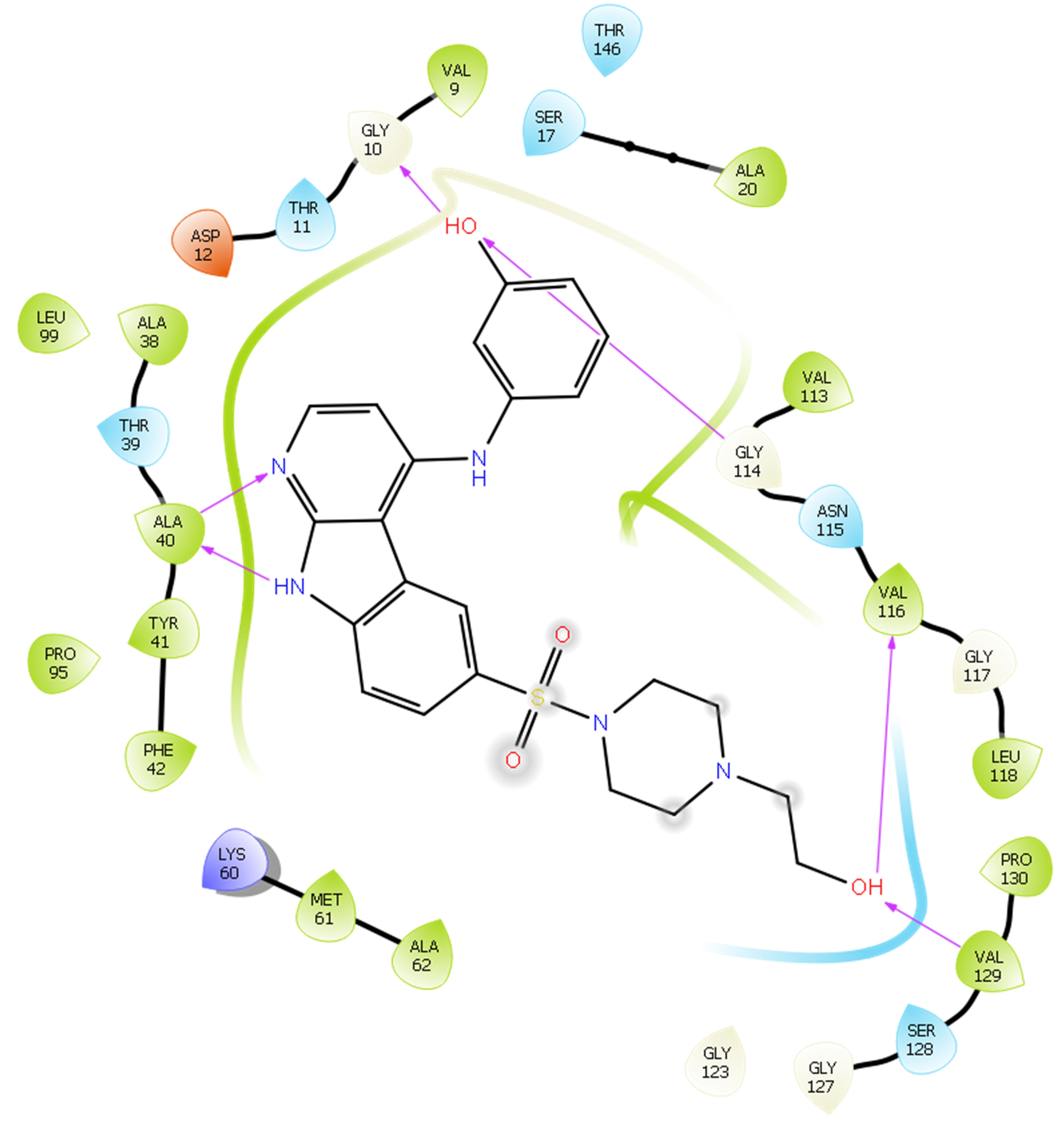

Supplement: Supplementary Figure 5 — 2D interaction map of docked pose of ChEMBL3133832 with the binding site residues of MSMEG_3811. For details related to color code, please refer to the legend to Supplementary Figure 3. [file Image_5.JPEG]

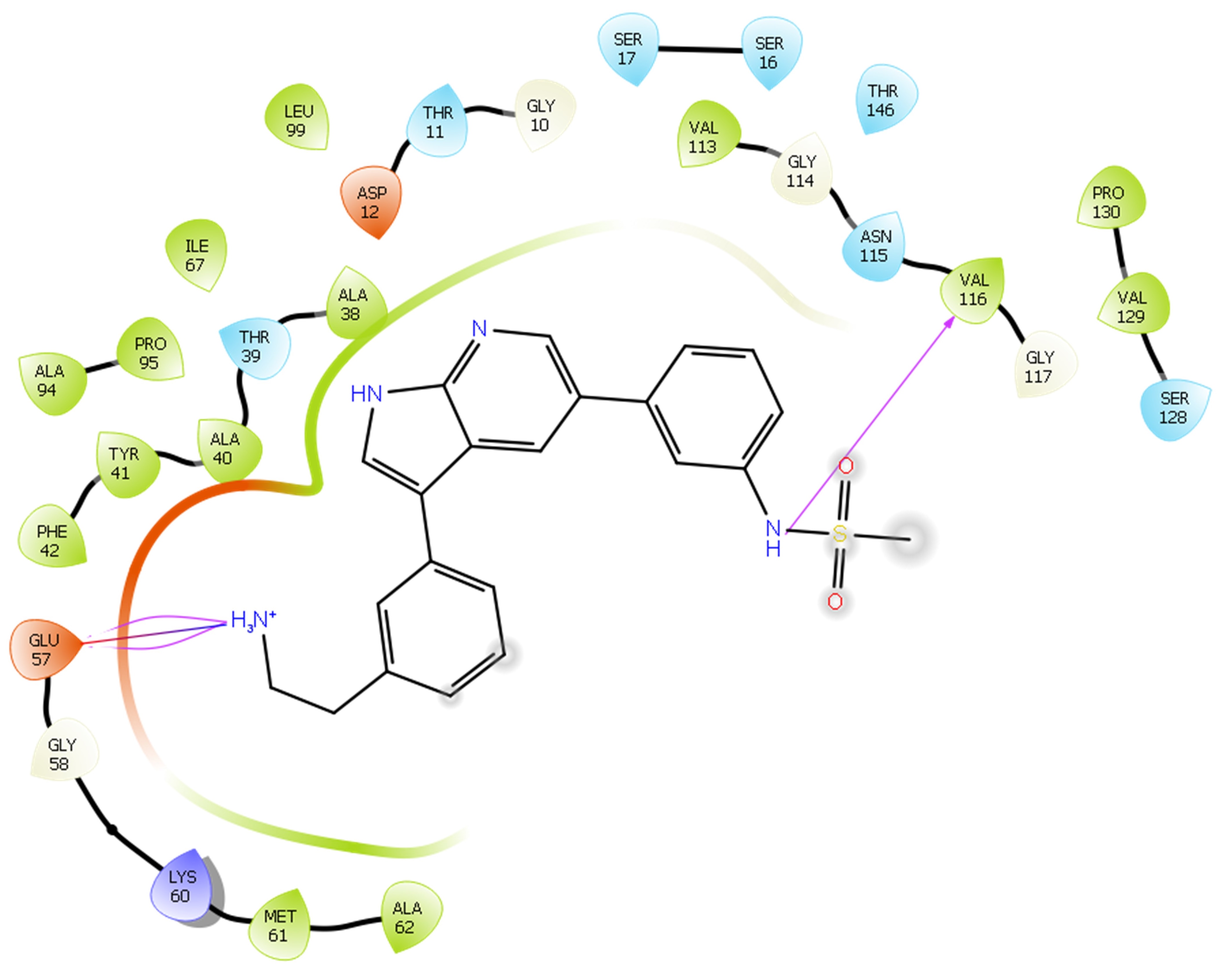

Supplement: Supplementary Figure 6 — 2D interaction map of docked pose of ChEMBL2109743 with the binding site residues of MSMEG_3811. For details related to color code, please refer to the legend to Supplementary Figure 3. [file Image_6.JPEG]

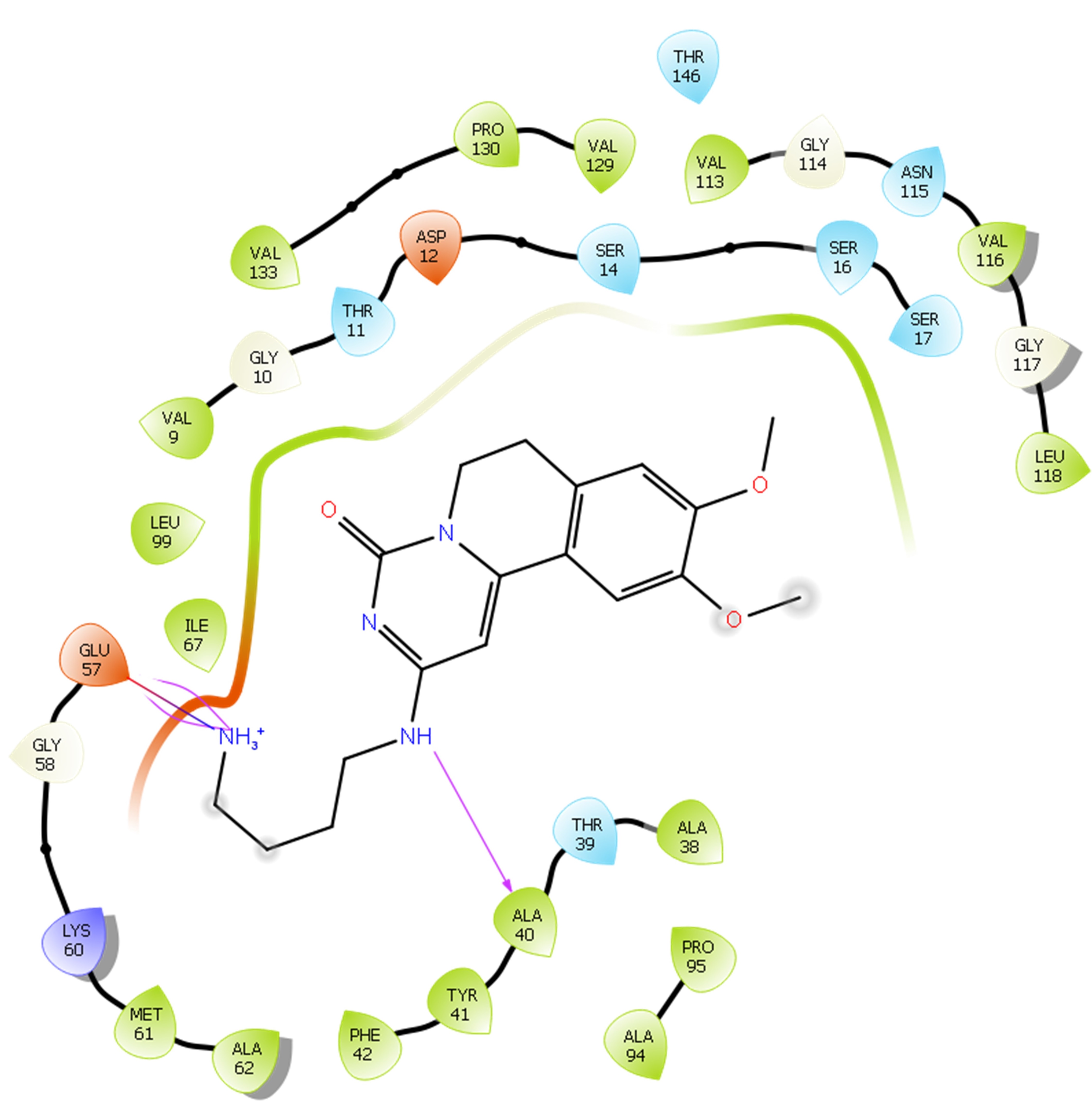

Supplement: Supplementary Figure 7 — 2D interaction map of docked pose of STOCK1N-42384 with the binding site residues of MSMEG_3811. For details related to color code, please refer to the legend to Supplementary Figure 3. [file Image_7.JPEG]

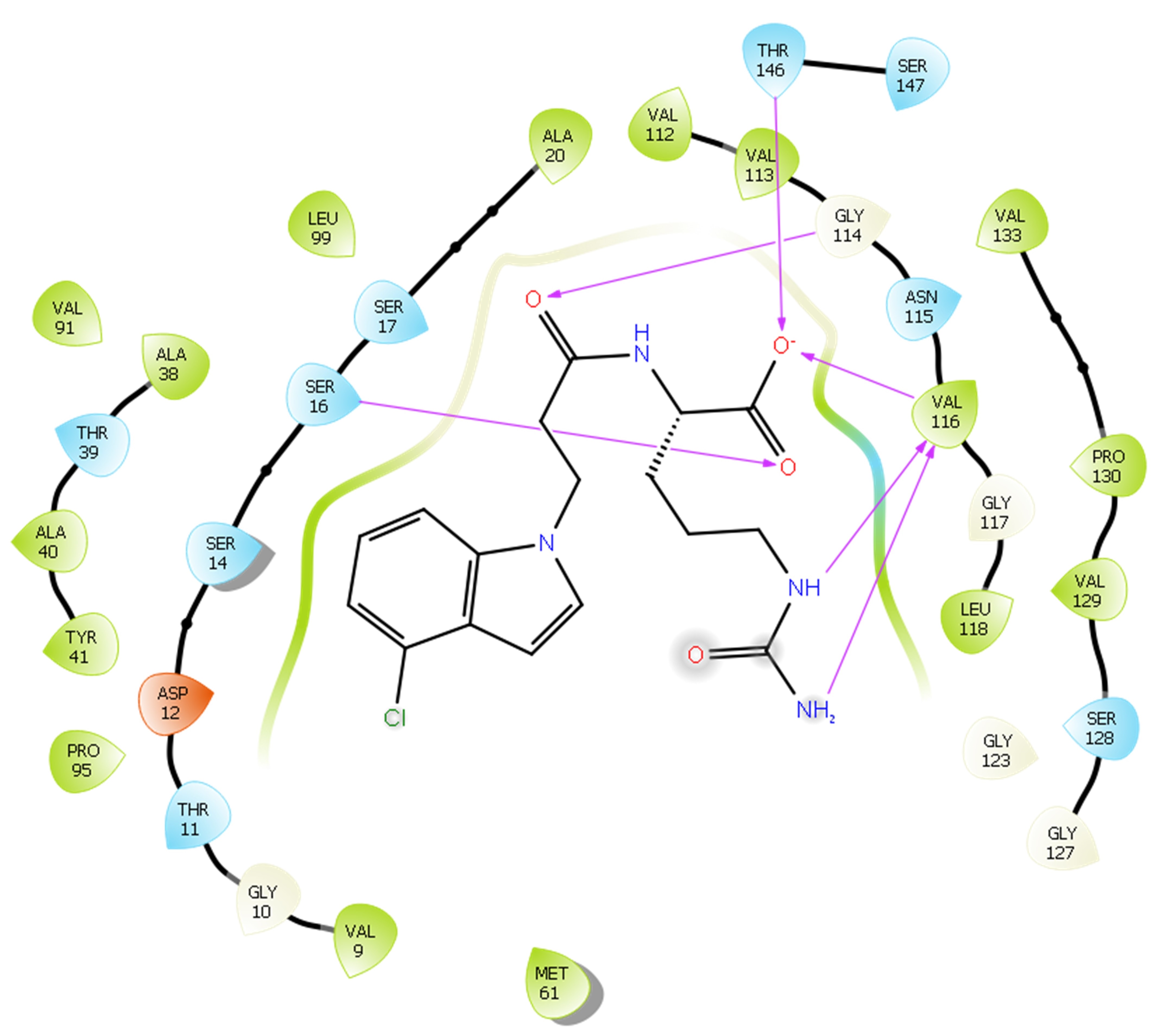

Supplement: Supplementary Figure 8 — 2D interaction map of docked pose of STOCK1N-74667 with the binding site residues of MSMEG_3811. For details related to color code, please refer to the legend to Supplementary Figure 3. [file Image_8.JPEG]

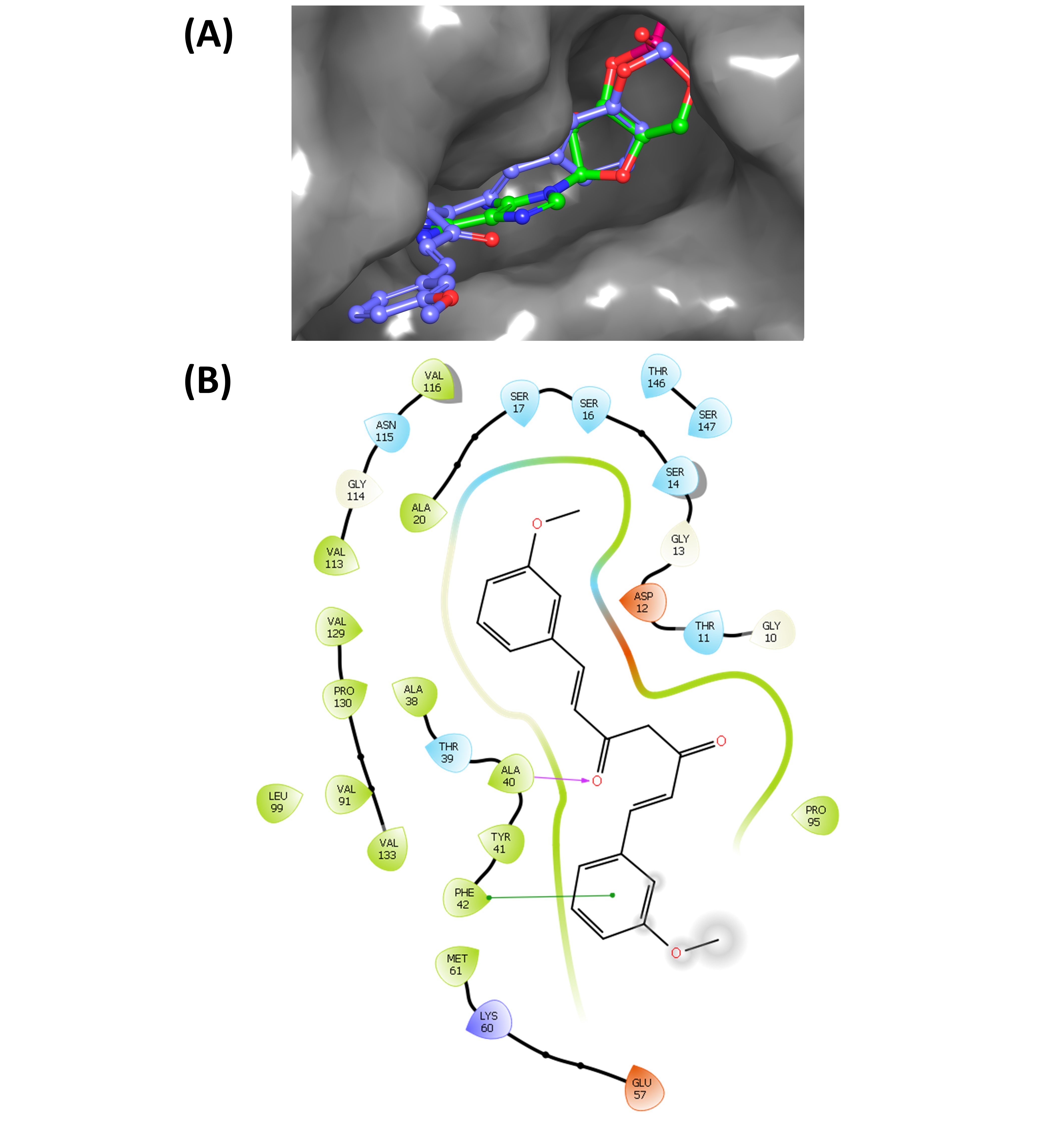

Supplement: Supplementary Figure 9 — Analysis of docked pose of Curcumin with the binding site residues of MSMEG_3811. (A) Overlay of docked pose of curcumin (violet stick) onto bound pose of cAMP (green stick). Nitrogen, and oxygen atoms are shown in blue and red, respectively. Hydrogen atoms were not displayed during image generation to maintain visual clarity. (B) 2D interaction map of docked pose of curcumin with the binding site residues of MSMEG_3811. For details related to color code, please refer to the legend to Supplementary Figure 3. [file Image_9.JPEG]

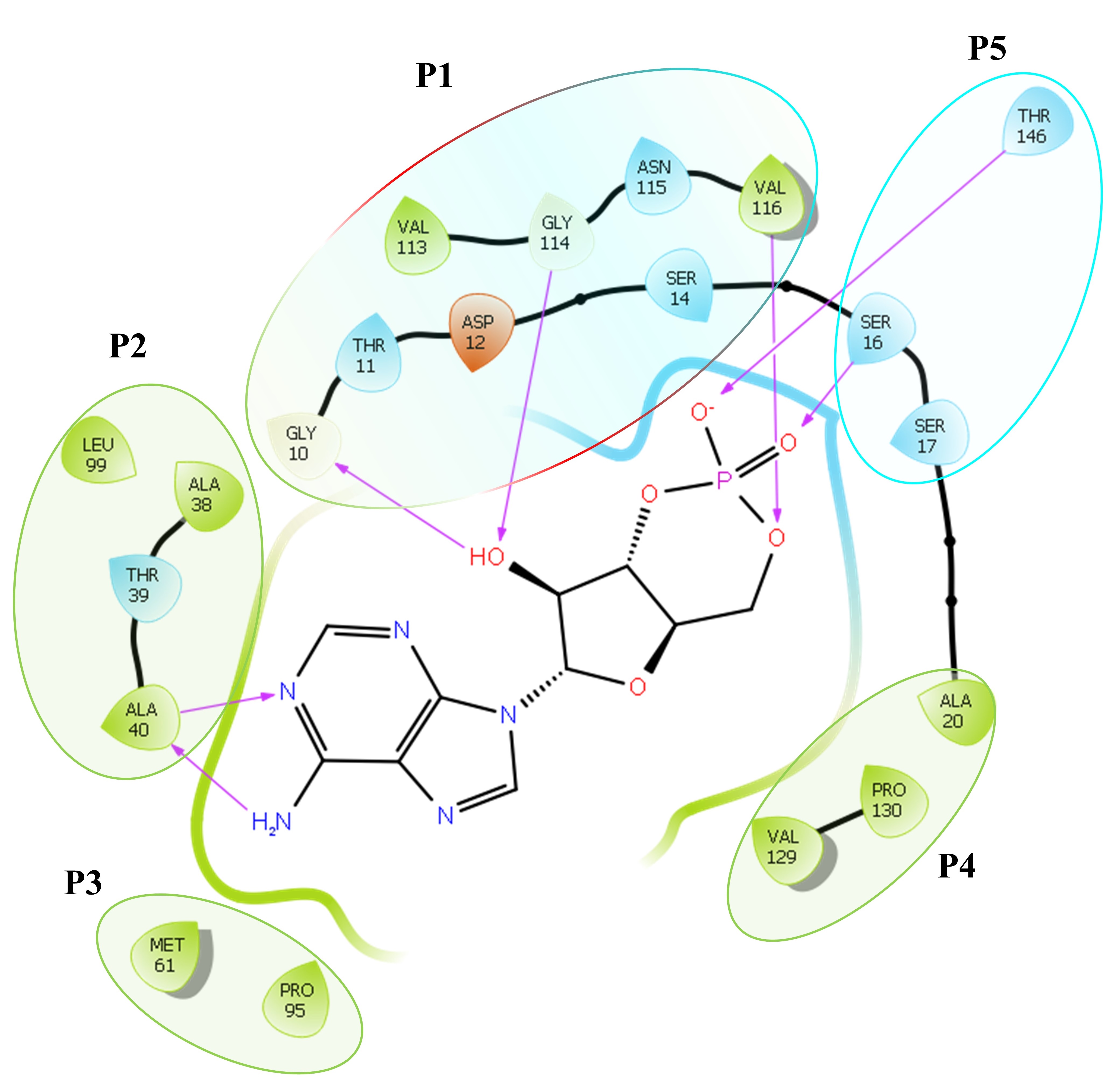

Supplement: Supplementary Figure 10 — Sub-pockets in cAMP binding site of MSMEG_3811 (PDB code: 5AHW). The five sub-pockets are marked P1, P2, P3, P4, and P5. The group of residues in each sub-pocket is encircled. For details related to color code, please refer to the legend to Supplementary Figure 3. [file Image_10.JPEG]
